# ﻿Five new epigean *Lagynochthonius* species (Pseudoscorpiones, Chthoniidae) from southern China

**DOI:** 10.3897/zookeys.1198.115609

**Published:** 2024-04-23

**Authors:** Jianzhou Sun, Xiangbo Guo, Feng Zhang

**Affiliations:** 1 Key Laboratory of Zoological Systematics and Application, College of Life Sciences, Hebei University, Baoding, Hebei 071002, China Hebei University Baoding China; 2 Hebei Basic Science Center for Biotic Interaction, Hebei University, Baoding, Hebei 071002, China Hebei University Baoding China

**Keywords:** Morphology, new species, pseudoscorpion, taxonomy

## Abstract

Five new *Lagynochthonius* species of the pseudoscorpion family Chthoniidae are described, based on morphological characters: *Lagynochthoniusduo***sp. nov.**, *Lagynochthoniusgibbus***sp. nov.**, *Lagynochthoniushepingensis***sp. nov.**, *Lagynochthoniushoui***sp. nov.**, and *Lagynochthoniussanhuaensis***sp. nov.** All specimens were collected from epigean habitats in southern China.

## ﻿Introduction

The genus *Lagynochthonius* Beier, 1951, belonging to the family Chthoniidae Daday, 1889, subfamily Chthoniinae Daday, 1889, tribe Tyrannochthoniini Chamberlin, 1962, was erected by Beier (1951) as a subgenus of *Tyrannochthonius* Chamberlin, 1929, subsequently elevated to generic status by Chamberlin (1962). It can be recognized by the trichobothria *ib* and *isb* situated close together in a median or sub-basal position on the dorsum of the chelal hand; the trichobothrium *sb* situated midway between *st* and *b*; the coxal spines commonly long and present only on coxae II; the chelal hand distally constricted (or flask-shaped), base of movable finger with strongly sclerotized apodeme and the modified tooth (*td*) of the fixed chelal finger displaced onto the prolateral-retrolateral face (Chamberlin 1962; Harvey 1989; Muchmore 1991; Judson 2007; Edward and Harvey 2008). The movable finger of *Tyrannochthinius* is only slightly sclerotized, and the fixed chelal finger does not have modified tooth (*td*), which are the most important differences between these two genera (Chamberlin 1962; Harvey 1989; Muchmore 1991).

*Lagynochthonius* pseudoscorpions usually live in litter layer or soil, under rocks and stones, in caves. At present, this chthoniid genus contains 79 species, of which 32 are distributed in China (Hou et al. 2023a; WPC 2024). Because the biodiversity of cave environments has received a high attention in recent years, most of the reported *Lagynochthonius* species from China are cave-dwelling (Li et al. 2019; Hou et al. 2022a, b, 2023a, b), and only seven species, *Lagynochthoniusbrachydigitatus* Zhang & Zhang, 2014, *L.harveyi* Zhang & Zhang, 2014, *L.leptopalpus* Hu & Zhang, 2012, *L.medog* Zhang & Zhang, 2014, *L.niger* Hu & Zhang, 2012, *L.sinensis* Beier, 1967 and *L.tonkinensis* Beier, 1951, are reported in epigean environments. In this study, five new species of *Lagynochthonius* are described, all of them collected from epigean habitats in southern China.

## ﻿Materials and methods

### ﻿Specimen preparation and examination

The specimens examined for this study are preserved in 75% ethyl alcohol in a refrigerator at -20 °C and deposited in the Museum of Hebei University (MHBU) (Baoding, China). Photographs and measurements were taken using a Leica M205A stereomicroscope equipped with a Leica DFC550 camera. Drawings was made using the Inkscape software (v. 1.0.2.0). Detailed examination was conducted with an Olympus BX53 general optical microscope. All images were edited and formatted using Adobe Photoshop 2017.

### ﻿Terminology

Terminology and measurements follow Chamberlin (1931) with some small modifications to the terminology of trichobothria (Harvey 1992; Judson 2007) and chelicera (Judson 2007). The chela and legs are measured in lateral view and others are taken in dorsal view. All measurements are given in mm unless noted otherwise. Proportions and measurements of chelicerae, carapace and pedipalps correspond to length/breadth, and those of legs to length/depth.

The following abbreviations are used in the text: for the chelal trichobothria: ***b*** = basal; ***sb*** = sub-basal; ***st*** = subterminal; ***t*** = terminal; ***ib*** = interior basal; ***isb*** = interior sub-basal; ***ist*** = interior sub-terminal; ***it*** = interior terminal; ***eb*** = exterior basal; ***esb*** = exterior sub-basal; ***est*** = exterior sub-terminal; ***et*** = exterior terminal. For other abbreviations: ***af***, apical sensilla of fixed chelal finger, ***am***, apical sensilla of movable chelal finger; ***dx***, duplex trichobothria; ***p_1–2_***, proximal sensilla of movable chelal finger; sc, microsetae (chemosensory setae); ***td***, modified tooth.

## ﻿Taxonomy

### ﻿Family Chthoniidae Daday, 1889

**Subfamily Chthoniinae Daday, 1889**

**Tribe Tyrannochthoniini Chamberlin, 1962**

#### ﻿Genus Lagynochthonius

Taxon classificationAnimaliaPseudoscorpionesChthoniidae

Beier, 1951

F25D6564-6CCE-53A7-A4BA-8D6B4B6B302E

##### Type species.

*Chthoniusjohni* Redikorzev, 1922, by original designation.

#### ﻿ Lagynochthonius duo sp. nov.

Taxon classificationAnimaliaPseudoscorpionesChthoniidae

FE29129A-3FC8-5853-9BBE-FA2BEB52F423

https://zoobank.org/C97FE3FA-C67E-47FF-B960-895E9F933AFE

**Figure 1.**
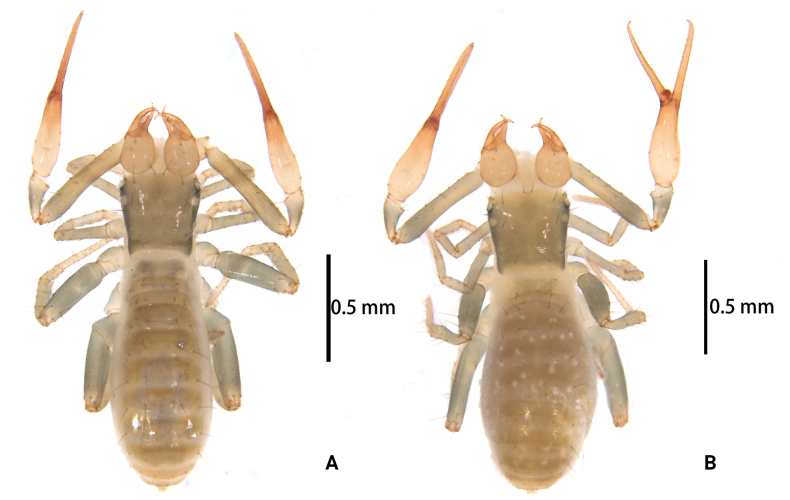
*Lagynochthoniusduo* sp. nov. **A** holotype male (dorsal view) **B** paratype female (dorsal view).

**Figure 2.**
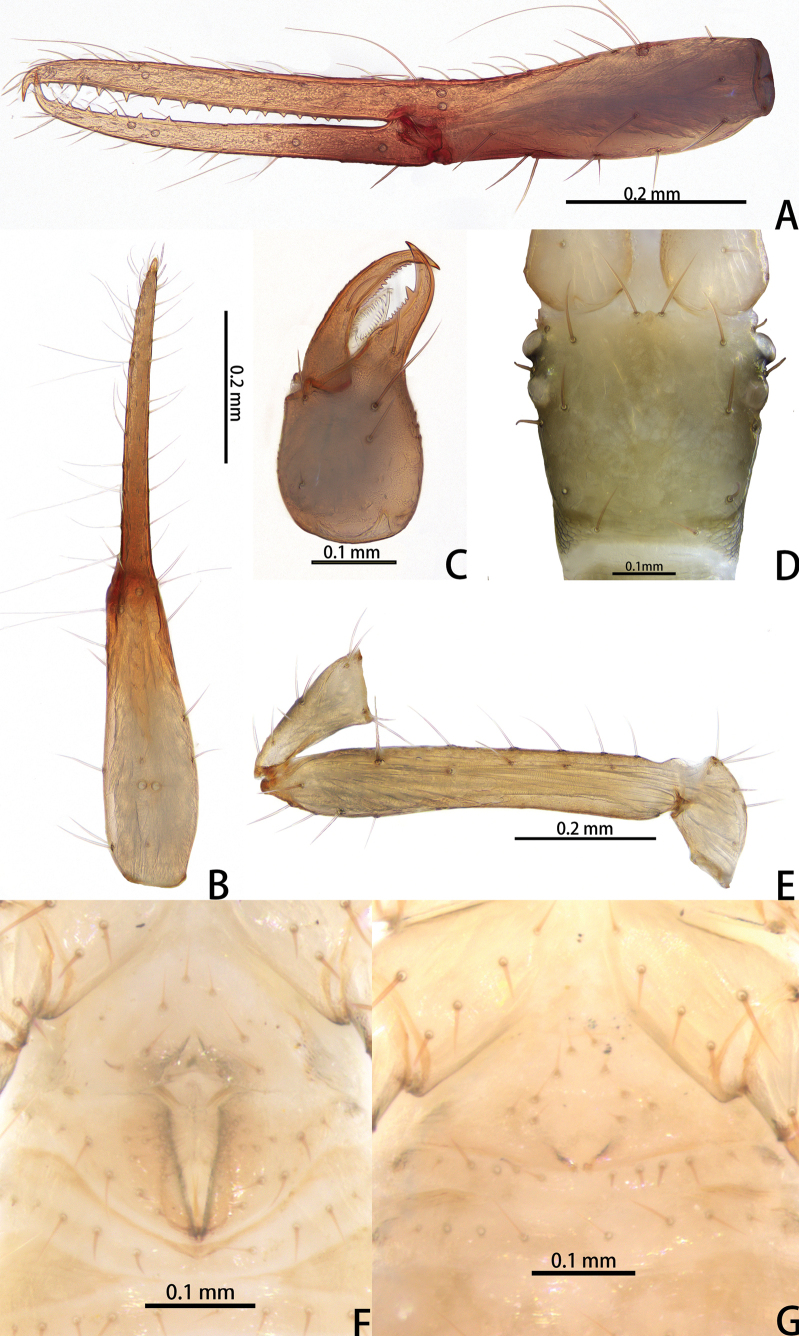
*Lagynochthoniusduo* sp. nov., holotype male (**A–F**) paratype female (**G**): **A** left chela (lateral view) **B** left chela (dorsal view) **C** right chelicera (dorsal view) **D** carapace (dorsal view) **E** Left pedipalp (minus chela, dorsal view) **F** male genital area (ventral view) **G** female genital area (ventral view).

**Figure 3.**
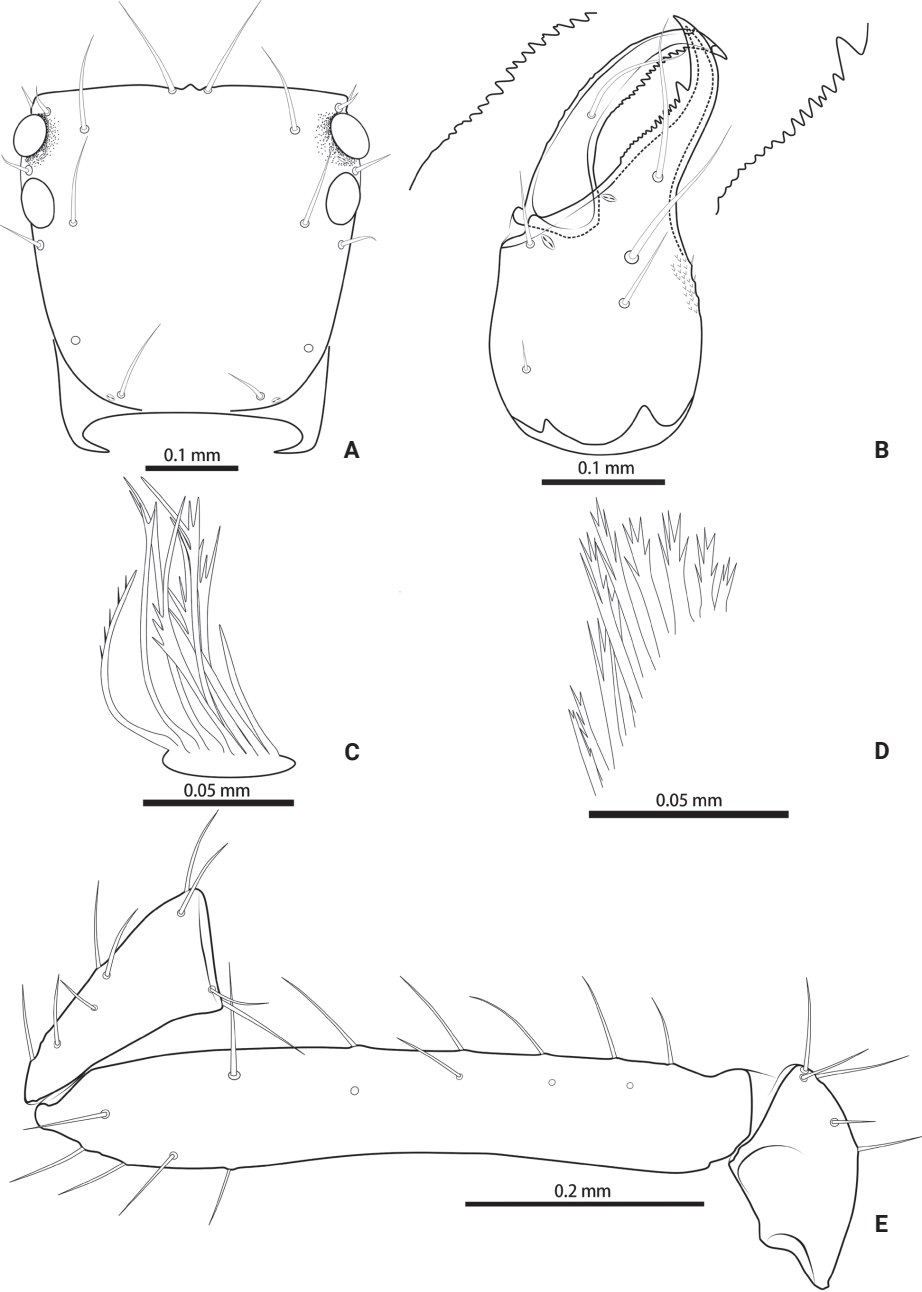
*Lagynochthoniusduo* sp. nov., holotype male **A** carapace (dorsal view) **B** left chelicera (dorsal view), with details of teeth **C** rallum **D** coxal spines on coxae II (ventral view) **E** left pedipalp (minus chela, dorsal view).

**Figure 4.**
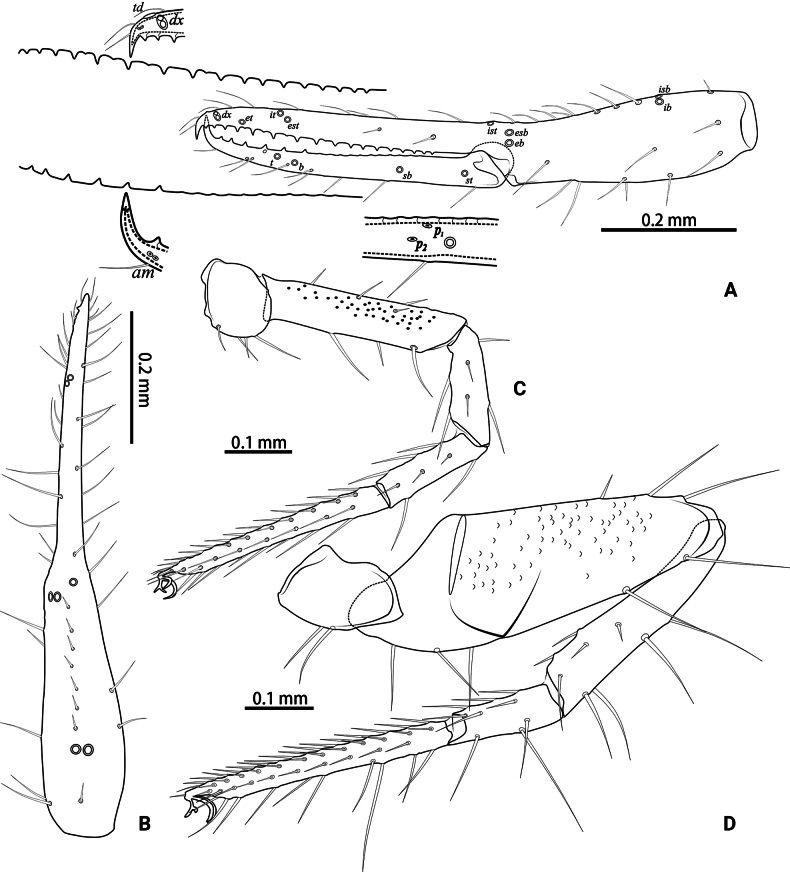
*Lagynochthoniusduo* sp. nov., holotype male **A** left chela (lateral view), with details of teeth and trichobothrial pattern **B** left chela (dorsal view) **C** Leg I (lateral view) **D** leg IV (lateral view). Abbreviations: for the chelal trichobothria: *b* = basal; *sb* = sub-basal; *st* = subterminal; *t* = terminal; *ib* = interior basal; *isb* = interior sub-basal; *ist* = interior sub-terminal; *it* = interior terminal; *eb* = exterior basal; *esb* = exterior sub-basal; *est* = exterior sub-terminal; *et* = exterior terminal. For other abbreviations: *af*, apical sensilla of fixed chelal finger, *am*, apical sensilla of movable chelal finger; *dx*, duplex trichobothria; *p_1–2_*, proximal sensilla of movable chelal finger; *td*, modified tooth.

**Figure 5.**
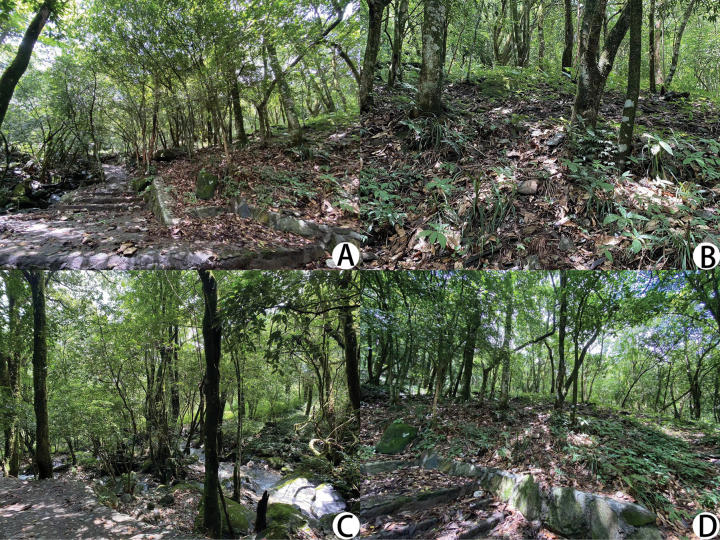
Type locality of *Lagynochthoniusduo* sp. nov. **A** stone step road **B** selected deciduous layers **C** beside the stream **D** areas where *L.duo* sp. nov. specimens were collected.

Figs 1 , 2 , 3 , 4 , 5 Chinese name: 双毛拉伪蝎

##### Type material.

***Holotype*** ♂ (Ps.-MHBU-GX2022080201): China, Guangxi, Guilin City, Longsheng Autonomous County, Longji Town, Anjiangping Area, under topsoil and in the leaf litter layer [25°42′15.15″N, 110°3′3.87″E], 419 m a.s.l., 2 August 2023, Kun Yu & Jianzhou Sun leg. ***Paratypes***: 3 ♂ (Ps.-MHBU-GX2022080203–05) and 1 ♀ (Ps.-MHBU-GX2022080202), all with the same data as the holotype.

##### Etymology.

The specific name is derived from the Latin word *duo*, meaning dual, which refers to the presence of two setae on both tergites I and II. A noun in apposition.

##### Diagnosis.

(♂♀). Moderately sized epigean species; carapace with four eyes, anterior margin smooth and epistome triangular; tergites I and II each with two setae, III and IV each with four setae. Rallum with eight blades. Pedipalps slender, chela 6.17–7.27 (♂), 5.06 (♀) × as long as broad; femur 5.60–6.67 (♂), 6.70 (♀) × as long as broad; only fixed chelal finger with intercalary teeth and a modified accessory tooth (*td*) on prolateral-retrolateral face; chemosensory setae (*sc*) present on dorsum of chelal hand; sensilla present.

##### Description.

**Males** (holotype and paratypes) (Figs 1A, 2A–F, 3, 4).

***Color*** generally pale yellow, chelicerae, carapace, pedipalps and tergites slightly darker.

***Cephalothorax*** (Figs 2D, 3A): carapace nearly subquadrate, 0.95–0.97 × as long as broad, weakly constricted basally; posterior region with squamous sculpturing laterally, other area smooth, without furrows; anterior margin smooth, without serrate; epistome small and triangular; four well-developed eyes; with 18 setae arranged s4s: 4: 4: 2: 2, most setae heavy, long and gently curved, anterolateral setae much shorter than others; with two pairs of lyrifissures, first pair situated middle to the setae of ocular row, second pair situated lateral to the sole pair of setae of posterior row. Manducatory process with two acuminate distal setae, anterior seta less than 1/2 length of medial seta; apex of coxa I with a rounded anteromedial process; coxae II with 10 or 11 terminally indented coxal spines on each side, set as an oblique and arc row, central spines slightly longer than the others (Fig. 3D); intercoxal tubercle absent; Chaetotaxy of coxae: P 3, I 3, II 4, III 5, IV 5.

***Chelicera*** (Figs 2C, 3B): almost as long as carapace, 1.76–1.94 × as long as broad; five setae and two lyrifissures (exterior condylar lyrifissure and exterior lyrifissure) present on hand, all setae acuminate, ventrobasal setae shorter than others; movable finger with one medial seta. Cheliceral palm has moderate wrinkle on both ventral and dorsal sides. Both fingers well provided with teeth, fixed finger with 11–17 teeth, distal one largest; movable finger with 13–18 retrorse contiguous small teeth; galea completely vestigial (Fig. 3B). Serrula exterior with 20 and serrula interior with 14 blades. Rallum with eight blades, the distal one longest and recumbent basally, with fine barbules and slightly set apart from the other blades, latter tightly grouped and with long pinnae, some of which are subdivided (Fig. 3C).

***Pedipalp*** (Figs 2A, B, E, 3E, 4A, B): trochanter 1.78–1.89, femur 5.60–6.67, patella 2.30–2.56, chela 6.17–7.27, hand 2.83–3.27 × as long as broad; femur 2.43–2.84 × as long as patella; movable chelal finger 1.11–1.27 × as long as hand and 0.55 × as long as chela. Setae generally long and acuminate. Chelal palm gradually constricted towards fingers, apodeme complex of movable chelal finger strongly sclerotized. Fixed chelal finger and hand with eight trichobothria, movable chelal finger with four trichobothria, *ib* and *isb* situated close together, submedially on dorsum of chelal hand; *eb*, *esb* and *ist* at base of fixed chelal finger; *esb* and *eb* at almost the same level and *ist* slightly distal to *esb*; *it* slightly distal to *est*, situated subdistally; *et* slightly near to tip of fixed chelal finger, close to chelal teeth; *dx* situated distal to *et*; *sb* slightly closer to *st* than to *b*; *b* and *t* situated subdistally, *t* situated at the same level as *it* and distal to *b*; *est* situated distal to *b* and close to *it* (Figs 2A, 4A). Fixed chelal finger with sensilla *af_1–2_* close together, near tip; movable chelal finger with four sensilla: *am_1–2_* near tip, *p_2_* slightly distad of *p_1_*, *p_1_* slightly distad of *sb* and very close to chelal teeth (Fig. 4A). Microsetae (chemosensory setae) present on dorsum of chelal hand (Figs 2B, 4B). Both chelal fingers with a row of teeth, spaced regularly along the margin, teeth smaller distally and proximally: fixed finger with 17 or 18 well-spaced, pointed teeth, plus three or four intercalary microdenticles, and a modified accessory tooth on prolateral-retrolateral face (*td*, slightly distal to *dx*); movable finger with six or seven well-spaced, pointed teeth, plus nine or ten vestigial, rounded, contiguous basal teeth.

***Opisthosoma***: generally typical, pleural membrane finely granulated. All tergites and sternites undivided; setae uniseriate and acuminate. Tergal chaetotaxy I–XII: 2: 2: 4: 4: 4: 4: 4–5: 5–7: 5–6: 4: T2T: 0. Sternal chaetotaxy IV–XII: 8–10: 10–12: 11–12: 10–11: 10–12: 10–12: 9: -: 2. Genital region: sternite II with 6–10 setae scattered on median area, genital opening slit-like, sternite III with 18–20 setae (Fig. 3F).

***Legs*** (Fig. 4C, D): fine granulation present on anterodorsal faces of trochanter IV, femur I; scale-like texture display on anterodorsal faces of femoropatella IV. Leg I: femur 1.71–1.94 × as long as patella; tarsus 1.88–2.13 × as long as tibia. Leg IV: femoropatella 2.65–2.81 × as long as deep; tibia 4.38–5.14 × as long as deep; with basal tactile setae on both tarsal segments: basitarsus 2.50–3.20 × as long as deep (TS = 0.35–0.53), telotarsus 9.25–12.00 × as long as deep and 2.29–2.47 × as long as basitarsus (TS = 0.23–0.33). Setae of leg I (trochanter to tibia) 3–5: 7–11: 6–7: 8–12, setae of leg IV (trochanter to basitarsus) 3: 3: 7–8: 8–9: 5–8. Arolium not divided, slightly shorter than the simple claws.

**Adult female** (paratype) (Figs 1B, 2G). Mostly same as males; tergal chaetotaxy I–XII: 2: 2: 4: 4: 4: 6: 6: 7: 7: 4: T2T: 0; sternal chaetotaxy IV–XII: 9: 12: 12: 10: 10: 11: 9: -: 2. Genital region: sternite II with 10 setae scattered on median area, sternite III with a row of 10 setae.

***Dimensions*** (length/breadth or, in the case of the legs, length/depth in mm; ratios in parentheses). Males: body length 1.24–1.44. Pedipalps: trochanter 0.16–0.17/0.09 (1.78–1.89), femur 0.56–0.60/0.09–0.10 (5.60–6.67), patella 0.21–0.24/0.09–0.10 (2.30–2.56), chela 0.74–0.80/0.11–0.12 (6.17–7.27), hand 0.34–0.37/0.11–0.12 (2.83–3.27), movable chelal finger length 0.41–0.44. Chelicera 0.29–0.33/0.16–0.17 (1.76–1.94), movable finger length 0.20–0.21. Carapace 0.34–0.36/0.35–0.38 (0.95–0.97). Leg I: trochanter 0.10–0.12/0.08 (1.25–1.50), femur 0.29–0.33/0.06 (4.83–5.50), patella 0.17/0.05–0.06 (2.83–3.40), tibia 0.15–0.17/0.04 (3.75–4.25), tarsus 0.32–0.35/0.04 (8.00–8.75). Leg IV: trochanter 0.15–0.17/0.10 (1.50–1.70), femoropatella 0.45–0.53/0.16–0.20 (2.65–2.81), tibia 0.33–0.36/0.06–0.08 (4.38–5.14), basitarsus 0.15–0.17/0.05–0.06 (2.50–3.20), telotarsus 0.36–0.39/0.03–0.04 (9.25–12.00).

**Females**: body length 1.57. Pedipalps: trochanter 0.17/0.11 (1.54), femur 0.67/0.10 (6.70), patella 0.26/0.11 (2.36), chela 0.91/0.18 (5.06), hand 0.44/0.18 (2.44), movable chelal finger length 0.49. Chelicera 0.37/0.21 (1.76), movable finger length 0.23. Carapace 0.38/0.43 (0.88). Leg I: trochanter 0.18/0.10 (1.80), femur 0.35/0.07 (5.00), patella 0.18/0.06 (3.00), tibia 0.18/0.06 (3.00), tarsus 0.39/0.04 (9.75). Leg IV: trochanter 0.19/0.11 (1.73), femoropatella 0.59/0.22 (2.68), tibia 0.38/0.08 (4.75), basitarsus 0.18/0.07 (2.57), telotarsus 0.42/0.04 (10.50).

##### Remarks.

*Lagynochthoniusduo* sp. nov. differs from all other epigean species of the genus *Lagynochthonius* from China except *L.medog* by the tergal chaetotaxy I–IV: 2: 2: 4: 4. It differs from *L.medog* in the presence of an epistome, which is absent in *L.medog*, and in the presence of only fixed chelal fingers with intercalary teeth in *L.duo* sp. nov., whereas both chelal fingers have intercalary teeth in *L.medog* (Beier 1951, 1967; Hu and Zhang 2012a, b; Zhang and Zhang 2014).

##### Distribution.

China (Guangxi).

#### ﻿ Lagynochthonius gibbus sp. nov.

Taxon classificationAnimaliaPseudoscorpionesChthoniidae

99C291BC-C7BF-5B21-BAE9-AABB9626B695

https://zoobank.org/133928D2-D809-499F-A7B7-BD05D648727D

**Figure 6.**
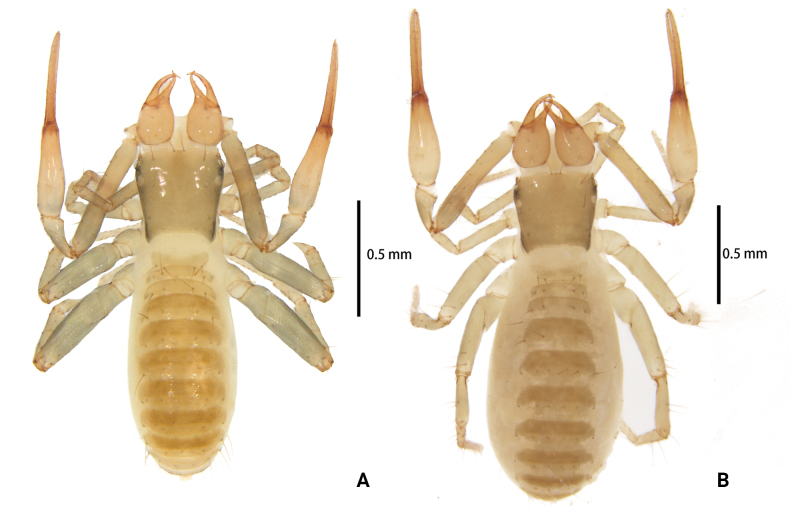
*Lagynochthoniusgibbus* sp. nov. **A** holotype male (dorsal view) **B** paratype female (dorsal view).

**Figure 7.**
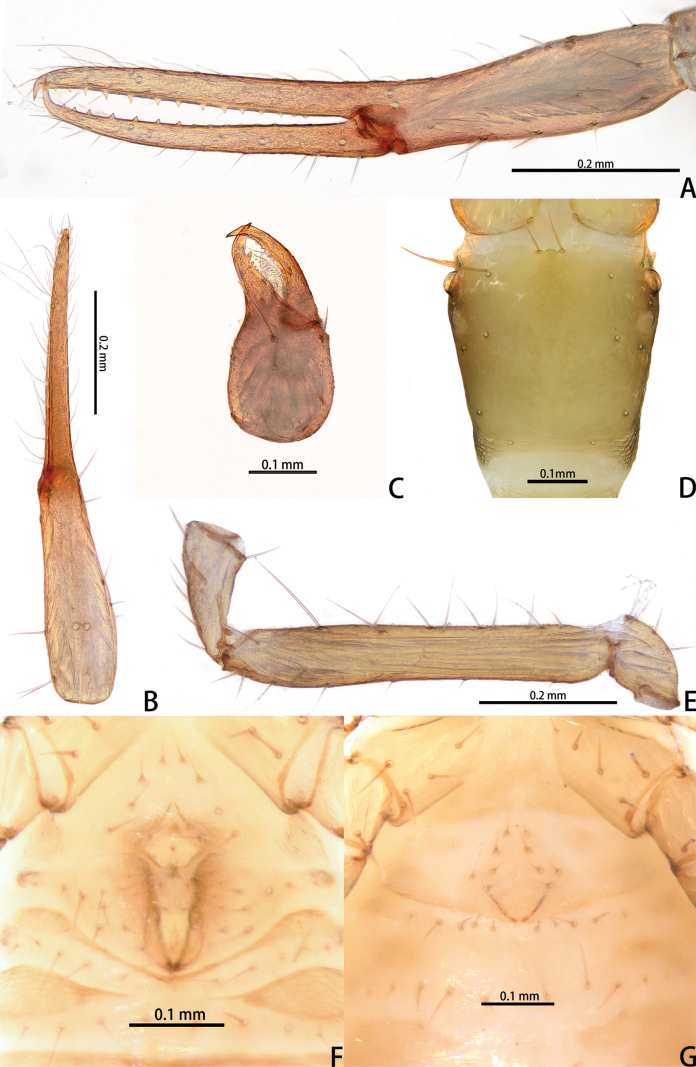
*Lagynochthoniusgibbus* sp. nov., holotype male (**A–F**) paratype female (**G**): **A** left chela (lateral view) **B** left chela (dorsal view) **C** right chelicera (dorsal view) **D** carapace (dorsal view) **E** left pedipalp (minus chela, dorsal view) **F** male genital area (ventral view) **G** female genital area (ventral view).

**Figure 8.**
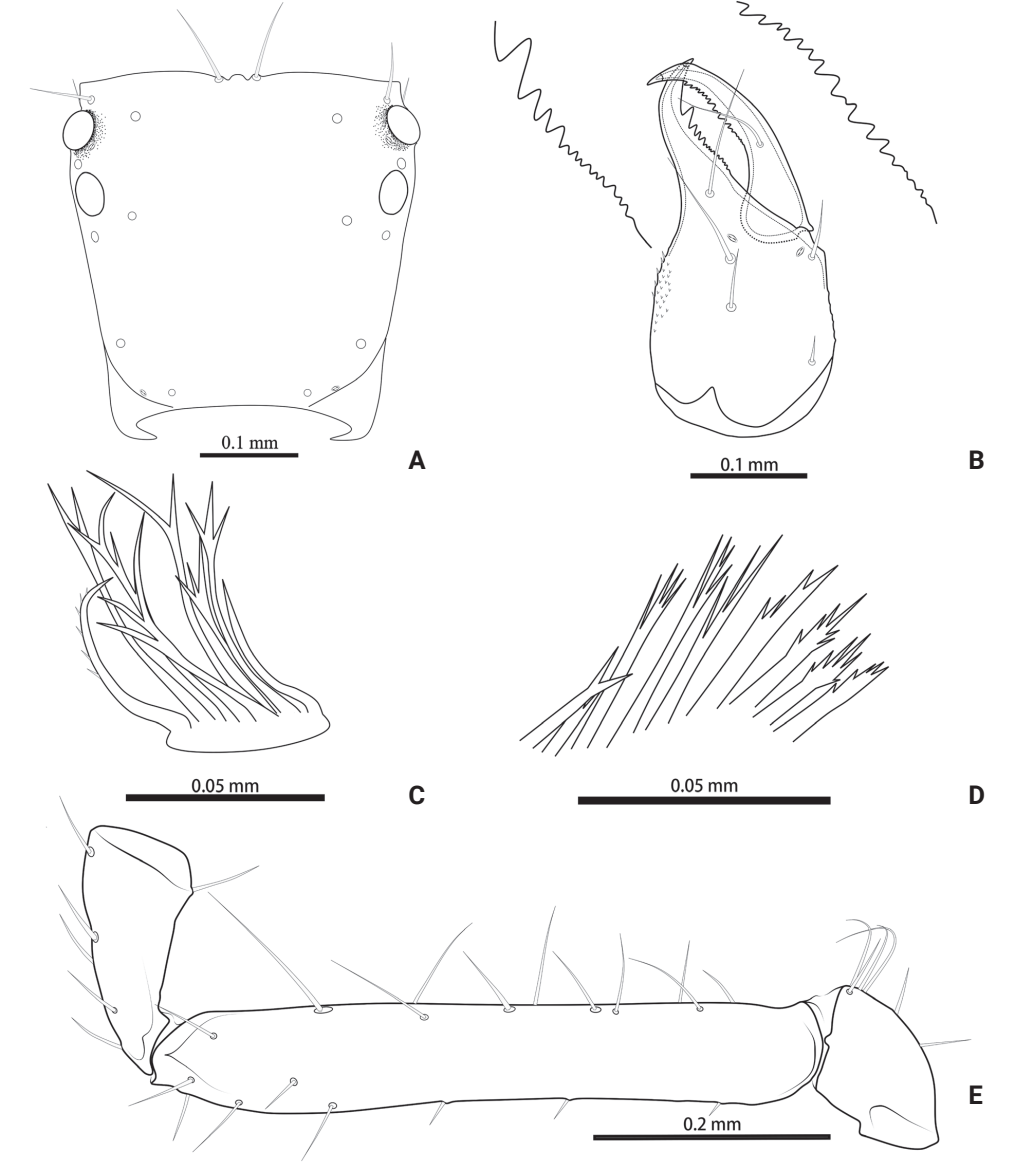
*Lagynochthoniusgibbus* sp. nov., holotype male **A** carapace (dorsal view) **B** right chelicera (dorsal view), with details of teeth **C** rallum **D** coxal spines on coxae II (ventral view) **E** left pedipalp (minus chela, dorsal view).

**Figure 9.**
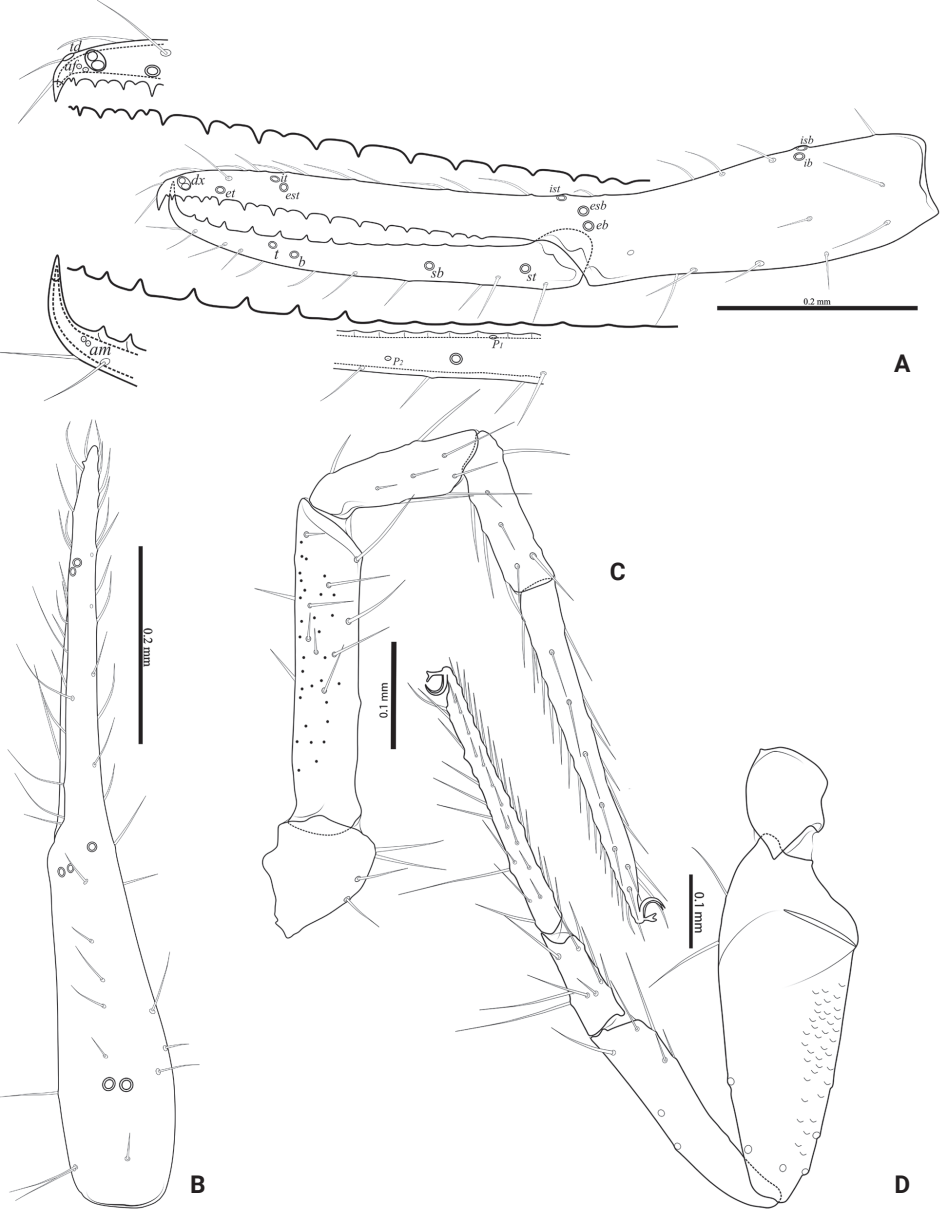
*Lagynochthoniusgibbus* sp. nov., holotype male **A** left chela (lateral view), with details of teeth and trichobothrial pattern **B** left chela (dorsal view) **C** leg I (lateral view) **D** leg IV (lateral view). Abbreviations: for the chelal trichobothria: *b* = basal; *sb* = sub-basal; *st* = subterminal; *t* = terminal; *ib* = interior basal; *isb* = interior sub-basal; *ist* = interior sub-terminal; *it* = interior terminal; *eb* = exterior basal; *esb* = exterior sub-basal; *est* = exterior sub-terminal; *et* = exterior terminal. For other abbreviations: *af*, apical sensilla of fixed chelal finger, *am*, apical sensilla of movable chelal finger; *dx*, duplex trichobothria; *p_1–2_*, proximal sensilla of movable chelal finger; *td*, modified tooth.

Figs 6 , 7 , 8 , 9 Chinese name: 驼峰拉伪蝎

##### Type material.

***Holotype*** ♂ (Ps.-MHBU-GZ2022070301): China, Guizhou Province, Tongren City, Jiangkou County, 500 m near Wanjiatun, under topsoil and in the leaf litter layer [27°43′32.5″N, 108°41′17.9″E], 509 m a.s.l., 3 July 2022, Yanmeng Hou, Lu Zhang, Nana Zhan, Jianzhou Sun & Long Lin leg. ***Paratype***: 1 ♀ (Ps.-MHBU-GZ2022070302), all with the same data as the holotype, 2 ♂ (Ps.-MHBU-GZ2022062805–06) and 2 ♀ (Ps.-MHBU-GZ2022062803–04): Tongren City, Sinan County, Zhangjiazhai Town, 700 m near Zhangjiaping, under topsoil and in the leaf litter layer [27°56′39.16″N, 108°4′21.8″E], 731 m a.s.l., 28 June 20, Yanmeng Hou, Lu Zhang, Nana Zhan, Jianzhou Sun & Long Lin leg.

##### Etymology.

The specific name is derived from the Latin word *gibbus*, meaning hump-shaped, which refers to the shape of epistome. A noun in apposition.

##### Diagnosis.

(♂♀). Moderately sized epigean species; carapace with four eyes, anterior margin smooth and epistome hump-shaped; tergites I–IV each with four setae. Rallum with eight blades. Pedipalps slender, chela 6.64–7.00 (♂), 5.12–5.69 (♀) × as long as broad; femur 5.78–7.00 (♂), 5.64–6.33 (♀) × as long as broad; only fixed chelal finger with intercalary teeth and a modified accessory tooth (*td*) on prolateral-retrolateral face; chemosensory setae (*sc*) present on dorsum of chelal hand; sensilla present.

##### Description.

**Males** (holotype and paratypes) (Figs 6A, 7A–F, 8, 9).

***Color*** generally pale yellow, chelicerae, carapace, pedipalps and tergites slightly darker.

***Cephalothorax*** (Figs 7D, 8A): carapace nearly subquadrate, 0.97–1.03 × as long as broad, weakly constricted basally; posterior region with squamous sculpturing laterally, other area smooth, without furrows; anterior margin smooth, without serrate; epistome small and hump-shaped; four eyes, anterior pair of eyes well-developed, posterior pair with flat lenses; with 18 setae arranged s4s: 4: 4: 2: 2, most setae heavy, long and gently curved, anterolateral setae much shorter than others; with two pairs of lyrifissures, first pair situated middle to the setae of ocular row, second pair situated lateral to the sole pair of setae of posterior row. Manducatory process with two acuminate distal setae, anterior seta less than 1/2 length of medial seta; apex of coxa I with a rounded anteromedial process; coxae II with 9–12 terminally indented coxal spines on each side, set as an oblique and arc row, central spines slightly longer than the others (Fig. 8D); intercoxal tubercle absent; Chaetotaxy of coxae: P 3, I 3, II 4, III 5, IV 5.

***Chelicera*** (Figs 7C, 8B): almost as long as carapace, 1.71–2.00 × as long as broad; five setae and two lyrifissures (exterior condylar lyrifissure and exterior lyrifissure) present on hand, all setae acuminate, ventrobasal setae shorter than others; movable finger with one medial seta. Cheliceral palm has moderate wrinkle on both ventral and dorsal sides. Both fingers well provided with teeth, fixed finger with 12–15 teeth, distal one largest; movable finger with 16–18 retrorse contiguous small teeth; galea completely vestigial (Fig. 8B). Serrula exterior with 17–20 and serrula interior with 14–20 blades. Rallum with eight blades, the distal one longest and recumbent basally, with fine barbules and slightly set apart from the other blades, latter tightly grouped and with long pinnae, some of which are subdivided (Fig. 8C).

***Pedipalp*** (Figs 7A, B, E, 8E, 9A, B): trochanter 1.78–1.89, femur 5.78–7.00, patella 2.33–2.56, chela 6.64–7.00, hand 3.09–3.18 × as long as broad; femur 2.43–2.60 × as long as patella; movable chelal finger 1.15–1.20 × as long as hand and 0.53–0.55 × as long as chela. Setae generally long and acuminate. Chelal palm gradually constricted towards fingers, apodeme complex of movable chelal finger strongly sclerotized. Fixed chelal finger and hand with eight trichobothria, movable chelal finger with four trichobothria, *ib* and *isb* situated close together, submedially on dorsum of chelal hand; *eb*, *esb* and *ist* forming a straight oblique row at base of fixed chelal finger; *it* slightly distal to *est*, situated subdistally; *et* slightly near to tip of fixed chelal finger, close to chelal teeth; *dx* situated distal to *et*; *sb* slightly closer to *st* than to *b*; *b* and *t* situated subdistally, *t* situated at the same level as *it* and distal to *b*; *est* situated distal to *b* and close to *it* (Figs 7A, 9A). Fixed chelal finger with sensilla *af_1–2_* close together, near tip; movable chelal finger with four sensilla: *am_1–2_* near tip, *p_2_* slightly distad of *sb*, *p_1_* proximad of *sb* and very close to chelal teeth (Fig. 9A). Microsetae (chemosensory setae) present on dorsum of chelal hand (Figs 7B, 9B). Both chelal fingers with a row of teeth, spaced regularly along the margin, teeth smaller distally and proximally: fixed finger with 20 or 21 well-spaced, pointed teeth, plus three or four intercalary microdenticles, and a modified accessory tooth on prolateral-retrolateral face (*td*, slightly distal to *dx*); movable finger with 6–8 well-spaced, pointed teeth, plus 5–7 vestigial, rounded and contiguous basal teeth.

***Opisthosoma***: generally typical, pleural membrane finely granulated. All tergites and sternites undivided; setae uniseriate and acuminate. Tergal chaetotaxy I–XII: 4: 4: 4: 4: 4: 4: 5–6: 6–7: 6: 4: T2T: 0. Sternal chaetotaxy IV–XII: 10–12: 12–16: 10–13: 12–13: 12–13: 11–12: 9–10: -: 2. Genital region: sternite II with eight setae scattered on median area, genital opening slit-like, sternite III with 18–24 setae (Fig. 7F).

***Legs*** (Fig. 9C, D): fine granulation present on anterodorsal faces of trochanter IV, femur I; scale-like texture display on anterodorsal faces of femoropatella IV. Leg I: femur 1.65–1.71 × as long as patella; tarsus 2.06–2.20 × as long as tibia. Leg IV: femoropatella 2.50–3.06 × as long as deep; tibia 4.43–4.71 × as long as deep; with basal tactile setae on both tarsal segments: basitarsus 2.33–3.40 × as long as deep (TS = 0.29–0.42), telotarsus 9.00–12.00 × as long as deep and 2.12–2.57 × as long as basitarsus (TS = 0.25–0.28). Setae of leg I (trochanter to tibia) 3–4: 7–11: 6–8: 8–9, setae of leg IV (trochanter to basitarsus) 3: 2–3: 4–7: 7–9: 6–10. Arolium not divided, slightly shorter than the simple claws.

**Adult females** (paratypes) (Figs 6B, 7G). Mostly same as males; tergal chaetotaxy I–XII: 4: 4: 4: 4–5: 5–6: 6: 5–6: 6: 5–8: 4–5: T2T: 0; sternal chaetotaxy IV–XII: 10–13: 11–13: 11–13: 10–14: 10–13: 10–13: 9–10: -: 2. Genital region: sternite II with 10 setae scattered on median area, sternite III with a row of 10–12 setae.

***Dimensions*** (length/breadth or, in the case of the legs, length/depth in mm; ratios in parentheses). Males: body length 1.23–1.39. Pedipalps: trochanter 0.16–0.17/0.09 (1.78–1.89), femur 0.52–0.56/0.08–0.09 (5.78–7.00), patella 0.20–0.23/0.08–0.09 (2.33–2.56), chela 0.73–0.77/0.11 (6.64–7.00), hand 0.34–0.35/0.11 (3.09–3.18), movable chelal finger length 0.39–0.42. Chelicera 0.29–0.30/0.15–0.17 (1.71–2.00), movable finger length 0.18–0.19. Carapace 0.33–0.35/0.34–0.35 (0.97–1.03). Leg I: trochanter 0.10–0.11/0.08–0.09 (1.11–1.38), femur 0.28–0.30/0.06 (4.67–5.00), patella 0.17–0.18/0.05 (3.40–3.60), tibia 0.15–0.16/0.04 (3.75–4.00), tarsus 0.33/0.03–0.04 (8.25–11.00). Leg IV: trochanter 0.10–0.17/0.10–0.11 (1.00–1.70), femoropatella 0.48–0.50/0.16–0.20 (2.50–3.06), tibia 0.31–0.33/0.07 (4.43–4.71), basitarsus 0.14–0.17/0.03–0.04 (2.33–3.40), telotarsus 0.36/0.03–0.04 (9.00–12.00).

**Females**: body length 1.64–1.82. Pedipalps: trochanter 0.16–0.19/0.09–0.11 (1.60–1.78), femur 0.57–0.64/0.09–0.11 (5.64–6.33), patella 0.23–0.26/0.08–0.12 (1.92–2.30), chela 0.79–0.91/0.15–0.17 (5.12–5.69), hand 0.38–0.43/0.15–0.17 (2.53–2.69), movable chelal finger length 0.42–0.48. Chelicera 0.34–0.38/0.19–0.22 (1.72–1.79), movable finger length 0.21–0.24. Carapace 0.35–0.40/0.39–0.44 (0.88–0.91). Leg I: trochanter 0.12–0.13/0.07–0.09 (1.33–1.71), femur 0.29–0.33/0.06–0.07 (4.71–5.50), patella 0.16–0.19/0.04–0.06 (2.67–4.75), tibia 0.15–0.18/0.04–0.05 (3.60–3.75), tarsus 0.34–0.37/0.04 (8.50–9.25). Leg IV: trochanter 0.16–0.17/0.10–0.14 (1.14–1.70), femoropatella 0.49–0.56/0.18–0.22 (2.52–2.72), tibia 0.33–0.37/0.06–0.08 (4.50–4.71), basitarsus 0.17–0.19/0.06 (2.83–3.17), telotarsus 0.36–0.43/0.03–0.04 (10.00–12.00).

##### Remarks.

*Lagynochthoniusgibbus* sp. nov. most closely resembles *L.duo* sp. nov. due to the presence of intercalary teeth only on the fixed chelal finger, the presence of eight blades on rallum, and similar size (chela length of males 0.73–0.80 mm, females 0.79–0.91 mm). However, the new species differs from *L.duo* sp. nov. in the shape of the epistome which is hump-shaped in *L.gibbus* sp. nov., but triangular in *L.duo* sp. nov., and in the number of setae on tergites I and II, with four setae on each in *L.gibbus* sp. nov. compared to two setae on each in *L.duo* sp. nov.

##### Distribution.

China (Guizhou Province).

#### ﻿ Lagynochthonius hepingensis sp. nov.

Taxon classificationAnimaliaPseudoscorpionesChthoniidae

3671072D-1623-5800-B7C8-3235F40920EF

https://zoobank.org/513D6885-A8B7-4527-9985-F14D9216A01F

**Figure 10.**
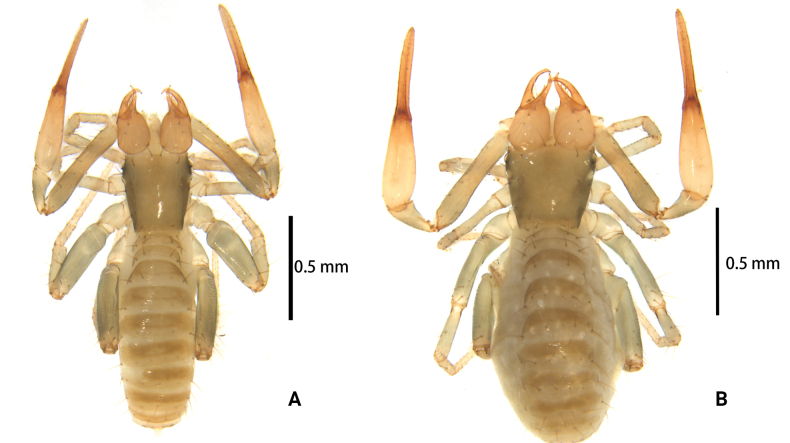
*Lagynochthoniushepingensis* sp. nov. **A** holotype male (dorsal view) **B** paratype female (dorsal view).

**Figure 11.**
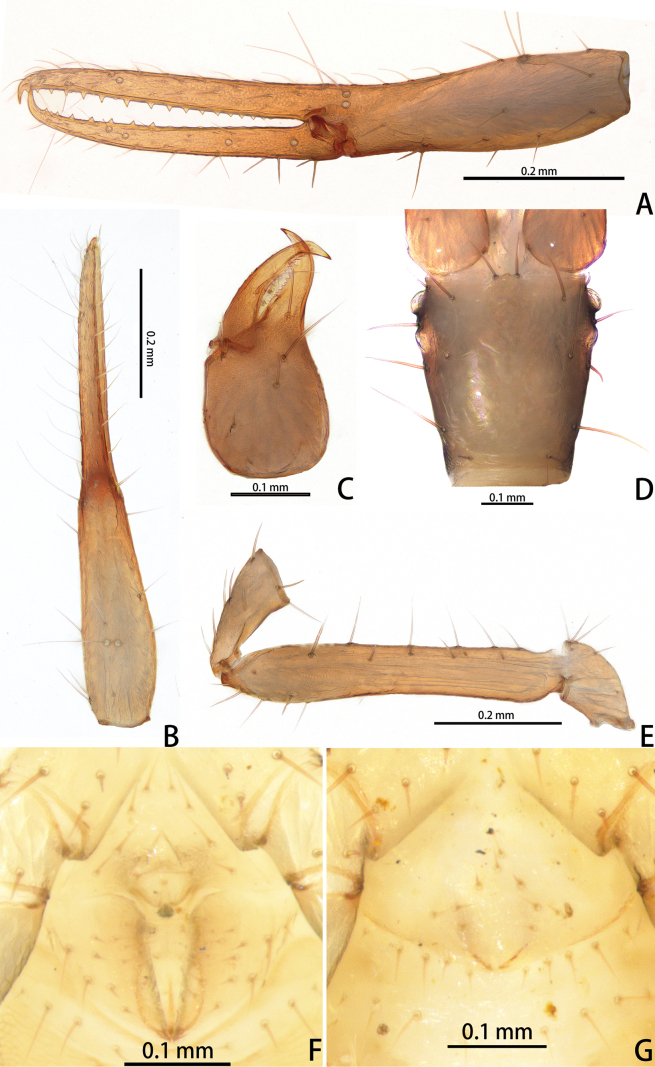
*Lagynochthoniushepingensis* sp. nov., holotype male (**A–F**) paratype female (**G**): **A** left chela (lateral view) **B** left chela (dorsal view) **C** left chelicera (dorsal view) **D** carapace (dorsal view) **E** left pedipalp (minus chela, dorsal view) **F** male genital area (ventral view) **G** female genital area (ventral view).

**Figure 12.**
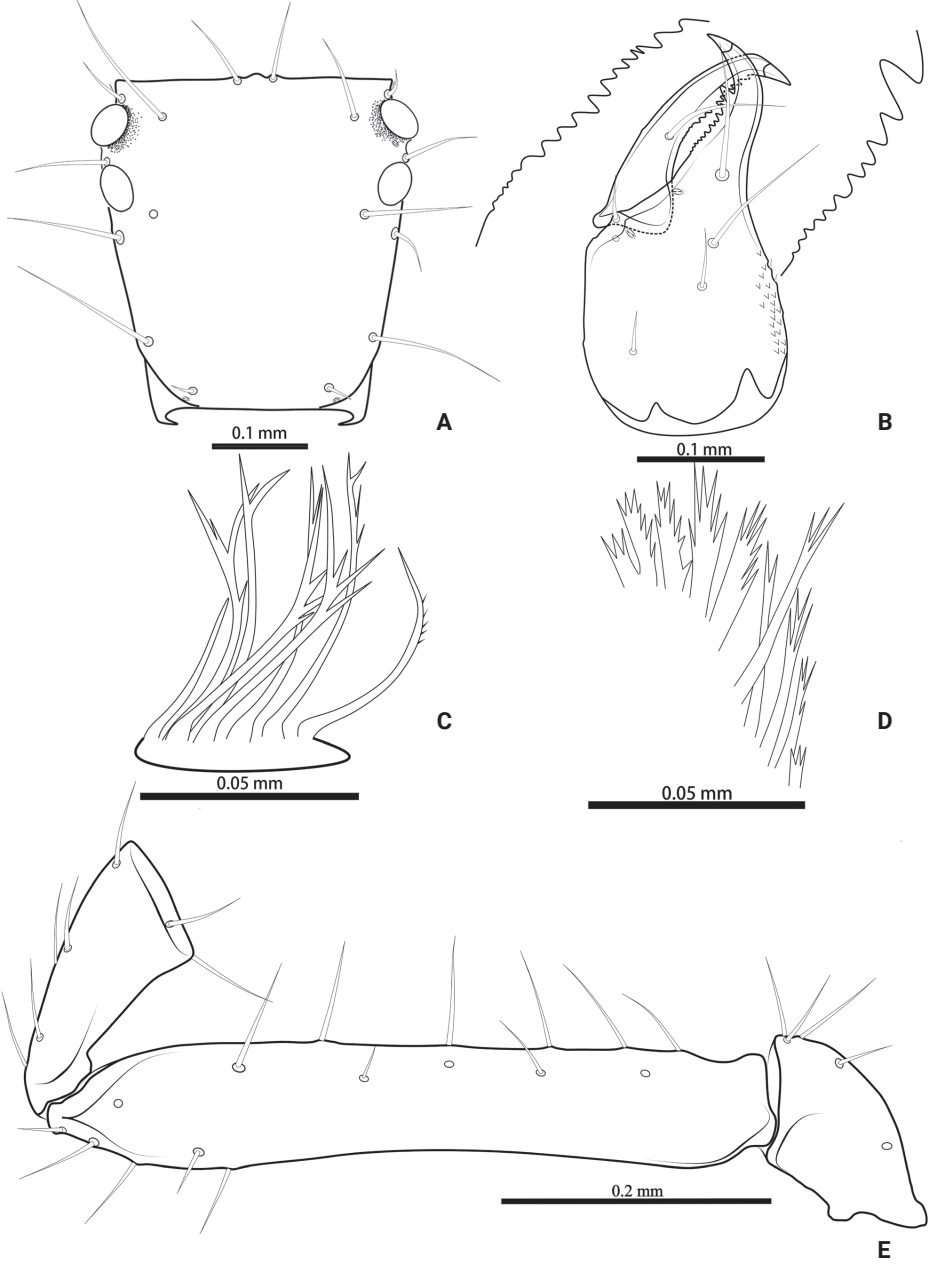
*Lagynochthoniushepingensis* sp. nov., holotype male **A** carapace (dorsal view) **B** right chelicera (dorsal view), with details of teeth **C** rallum **D** coxal spines on coxae II (ventral view) **E** left pedipalp (minus chela, dorsal view).

**Figure 13.**
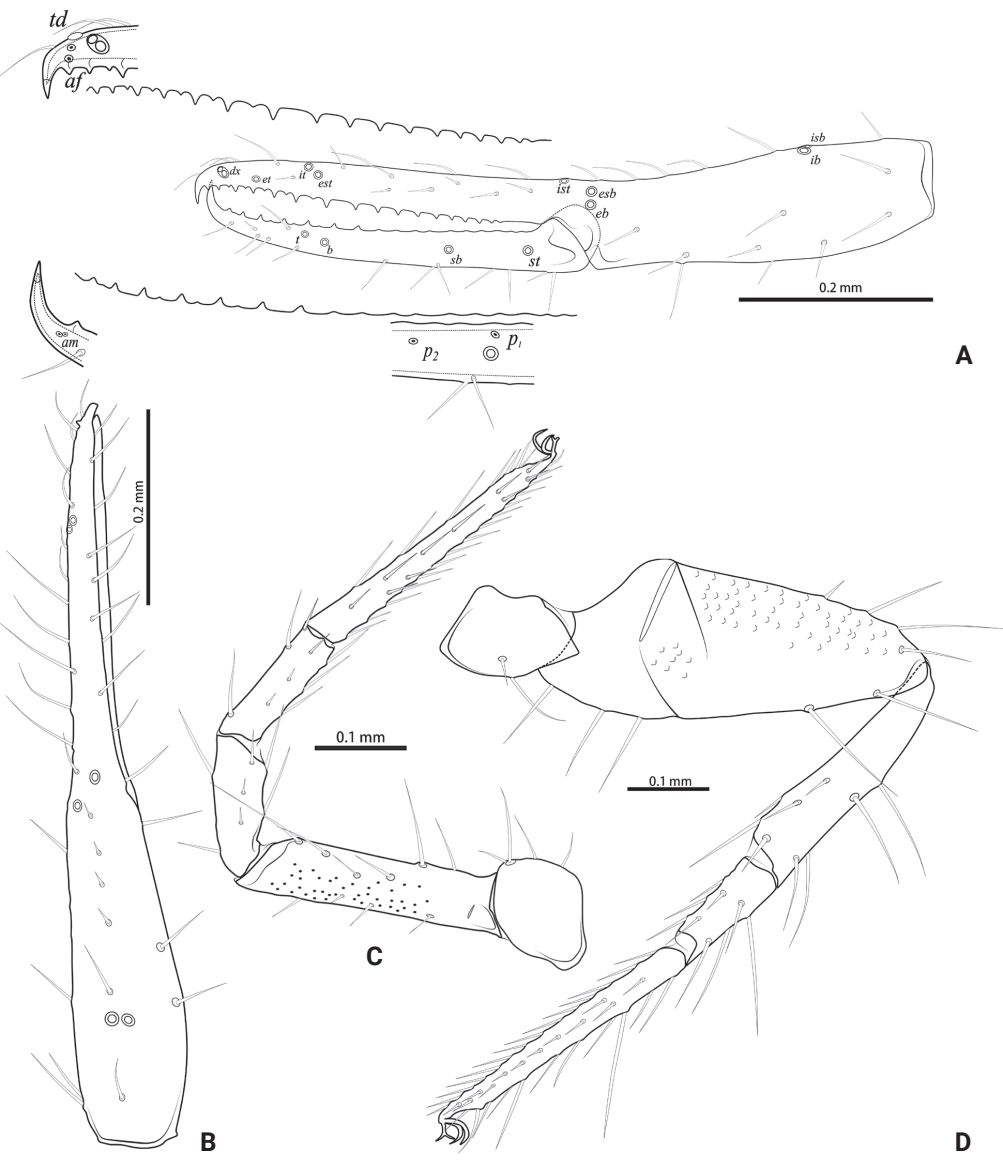
*Lagynochthoniushepingensis* sp. nov., holotype male **A** left chela (lateral view), with details of teeth and trichobothrial pattern **B** left chela (dorsal view) **C** leg I (lateral view) **D** leg IV (lateral view). Abbreviations: for the chelal trichobothria: *b* = basal; *sb* = sub-basal; *st* = subterminal; *t* = terminal; *ib* = interior basal; *isb* = interior sub-basal; *ist* = interior sub-terminal; *it* = interior terminal; *eb* = exterior basal; *esb* = exterior sub-basal; *est* = exterior sub-terminal; *et* = exterior terminal. For other abbreviations: *af*, apical sensilla of fixed chelal finger, *am*, apical sensilla of movable chelal finger; *dx*, duplex trichobothria; *p_1–2_*, proximal sensilla of movable chelal finger; *td*, modified tooth.

Figs 10 , 11 , 12 , 13 Chinese name: 和平拉伪蝎

##### Type material.

***Holotype*** ♂ (Ps.-MHBU-GZ2022080701): China, Guizhou Province, Qianxinan Prefecture, Wangmu County, Dayi Town, Heping Village, Near Provincial Highway 209, under topsoil and in the leaf litter layer [25°23′54.8″N, 106°7′37.08″E], 1553 m a.s.l., 7 August 2022, Yanmeng Hou, Lu Zhang, Jianzhou Sun & Wenlong Fan leg. ***Paratypes***: 5 ♂ (Ps.-MHBU-GZ2022080702–06) and 2 ♀ (Ps.-MHBU-GZ2022080707–08), all with the same data as the holotype.

##### Etymology.

Named after the Heping Village, the type locality. A noun in apposition.

##### Diagnosis.

(♂♀). Moderately sized epigean species; carapace with four eyes, anterior margin smooth and epistome hump-shaped; tergites I–IV each with four setae. Rallum with seven blades. Pedipalps slender, chela 6.08–6.82 (♂), 5.33–5.44 (♀) × as long as broad; femur 6.25–7.00 (♂), 6.20–6.30 (♀) × as long as broad; both chelal fingers with intercalary teeth, fixed chelal finger with a modified accessory tooth (*td*) on prolateral-retrolateral face; chemosensory setae (*sc*) present on dorsum of chelal hand; sensilla present.

##### Description.

**Males** (holotype and paratypes) (Figs 10A, 11A–F, 12, 13).

***Color*** generally pale yellow, chelicerae, carapace, pedipalps and tergites slightly darker.

***Cephalothorax*** (Figs 11D, 12A): carapace nearly subquadrate, 0.97–1.03 × as long as broad, strongly constricted basally; posterior region with squamous sculpturing laterally, other area smooth, without furrows; anterior margin smooth, without serrate; epistome small and hump-shaped; four well-developed eyes; with 18 setae arranged s4s: 4: 4: 2: 2, most setae heavy, long and gently curved, anterolateral setae much shorter than others; with two pairs of lyrifissures, first pair situated middle to the setae of ocular row, second pair situated lateral to the sole pair of setae of posterior row. Manducatory process with two acuminate distal setae, anterior seta less than 1/2 length of medial seta; apex of coxa I with a rounded anteromedial process; coxae II with 8–10 terminally indented coxal spines on each side, set as an oblique and arc row, central spines slightly longer than the others (Fig. 12D); intercoxal tubercle absent; Chaetotaxy of coxae: P 3, I 3, II 4, III 5, IV 5.

***Chelicera*** (Figs 11C, 12B): almost as long as carapace, 1.63–1.81 × as long as broad; five setae and two lyrifissures (exterior condylar lyrifissure and exterior lyrifissure) present on hand, all setae acuminate, ventrobasal setae shorter than others; movable finger with one medial seta. Cheliceral palm has moderate wrinkle on both ventral and dorsal sides. Both fingers well provided with teeth, fixed finger with 9–12 teeth, distal one largest; movable finger with 12–15 retrorse contiguous small teeth; galea completely vestigial (Fig. 12B). Serrula exterior with 16–23 and serrula interior with 14–20 blades. Rallum with eight blades, the distal one longest and recumbent basally, with fine barbules and slightly set apart from the other blades, latter tightly grouped and with long pinnae, some of which are subdivided (Fig. 12C).

***Pedipalp*** (Figs 11A, B, 11E, 12E, 13A, B): trochanter 1.50–1.78, femur 6.25–7.00, patella 2.11–2.88, chela 6.08–6.82, hand 2.83–3.10 × as long as broad; femur 2.43–2.84 × as long as patella; movable chelal finger 1.11–1.27 × as long as hand and 0.53–0.57 × as long as chela. Setae generally long and acuminate. Chelal palm gradually constricted towards fingers, apodeme complex of movable chelal finger strongly sclerotized. Fixed chelal finger and hand with eight trichobothria, movable chelal finger with four trichobothria, *ib* and *isb* situated close together, submedially on dorsum of chelal hand; *eb*, *esb* and *ist* at base of fixed chelal finger; *esb* and *eb* at almost the same level and *ist* slightly distal to *esb*; *it* slightly distal to *est*, situated subdistally; *et* slightly near to tip of fixed chelal finger, close to chelal teeth; *dx* situated distal to *et*; *sb* slightly closer to *st* than to *b*; *b* and *t* situated subdistally, *t* situated at the same level as *it* and distal to *b*; *est* situated distal to *b* and close to *it* (Figs 11A, 13A). Fixed chelal finger with sensilla *af_1–2_* close together, near tip; movable chelal finger with four sensilla: *am_1–2_* near tip, *p_2_* slightly distad of *sb*, *p_1_* proximad of *sb* and very close to chelal teeth (Fig. 13A). Microsetae (chemosensory setae) present on dorsum of chelal hand (Figs 11B, 13B). Both chelal fingers with a row of teeth, spaced regularly along the margin, teeth smaller distally and proximally: fixed finger with 16–20 well-spaced, pointed teeth, plus 5–7 intercalary microdenticles, and a modified accessory tooth on prolateral-retrolateral face (*td*, slightly distal to *dx*); movable finger with seven or eight well-spaced, pointed teeth, plus 2–5 intercalary microdenticles and six or seven vestigial, rounded and contiguous basal teeth.

***Opisthosoma***: generally typical, pleural membrane finely granulated. All tergites and sternites undivided; setae uniseriate and acuminate. Tergal chaetotaxy I–XII: 4: 4: 4: 4: 4: 4–5: 5–6: 5–7: 5–6: 4: T2T: 0. Sternal chaetotaxy IV–XII: 8–12: 10–12: 10–13: 10–12: 11–12: 10–12: 9: -: 2. Genital region: sternite II with 10 setae scattered on median area, genital opening slit-like, sternite III with 16–18 setae (Fig. 11F).

***Legs*** (Fig. 13C, D): fine granulation present on anterodorsal faces of trochanter IV, femur I; scale-like texture display on anterodorsal faces of femoropatella IV. Leg I: femur 1.69–2.00 × as long as patella; tarsus 1.93–2.36 × as long as tibia. Leg IV: femoropatella 2.40–2.67 × as long as deep; tibia 4.00–4.71 × as long as deep; with basal tactile setae on both tarsal segments: basitarsus 2.50–2.80 × as long as deep (TS = 0.33–0.43), telotarsus 8.75–11.67 × as long as deep and 2.33–2.64 × as long as basitarsus (TS = 0.24–0.30). Setae of leg I (trochanter to tibia) 3–4: 8–10: 6–7: 7–10, setae of leg IV (trochanter to basitarsus) 2–3: 3–4: 6–8: 7–9: 6–10. Arolium not divided, slightly shorter than the simple claws.

**Adult females** (paratypes) (Figs 10B, 11G). Mostly same as males; tergal chaetotaxy I–XII: 4: 4: 4: 4–5: 5–6: 6: 6: 6–8: 6: 4: T2T: 0; sternal chaetotaxy IV–XII: 10–12: 12–13: 12–14: 11–12: 10–12: 8–11: 10: -: 2. Genital region: sternite II with 10 setae scattered on median area, sternite III with a row of 12 setae.

***Dimensions*** (length/breadth or, in the case of the legs, length/depth in mm; ratios in parentheses). Males: body length 1.18–1.38. Pedipalps: trochanter 0.14–0.16/0.08–0.10 (1.50–1.78), femur 0.50–0.56/0.08–0.09 (6.25–7.00), patella 0.19–0.23/0.08–0.09 (2.11–2.88), chela 0.67–0.75/0.10–0.12 (6.08–6.82), hand 0.31–0.35/0.10–0.12 (2.83–3.10), movable chelal finger length 0.37–0.42. Chelicera 0.26–0.32/0.16–0.18 (1.63–1.81), movable finger length 0.15–0.18. Carapace 0.31–0.37/0.34–0.38 (0.89–1.09). Leg I: trochanter 0.10–0.12/0.07–0.10 (1.20–1.57), femur 0.26–0.30/0.05–0.06 (4.50–6.00), patella 0.15–0.16/0.05–0.06 (2.67–3.20), tibia 0.13–0.16/0.04 (3.25–4.00), tarsus 0.29–0.33/0.03–0.04 (7.25–11.00). Leg IV: trochanter 0.14–0.18/0.10–0.11 (1.36–1.63), femoropatella 0.44–0.50/0.17–0.20 (2.40–2.67), tibia 0.29–0.33/0.07–0.08 (4.00–4.71), basitarsus 0.13–0.15/0.05–0.06 (2.50–2.80), telotarsus 0.34–0.37/0.03–0.04 (8.75–11.67).

**Females**: body length 1.34–1.43. Pedipalps: trochanter 0.15–0.18/0.11–0.12 (1.25–1.64), femur 0.63–0.64/0.10 (6.20–6.30), patella 0.24–0.25/0.11–0.12 (2.08–2.18), chela 0.83–0.87/0.15–0.16 (5.33–5.44), hand 0.41–0.44/0.15–0.16 (2.73–7.75), movable chelal finger length 0.51–0.52. Chelicera 0.34–0.39/0.20 (1.70–1.95), movable finger length 0.21–0.23. Carapace 0.38–0.39/0.42 (0.90–0.93). Leg I: trochanter 0.11–0.13/0.09–0.10 (1.10–1.44), femur 0.32/0.06–0.07 (4.57–5.33), patella 0.16–0.17/0.06 (2.67–2.83), tibia 0.16–0.17/0.05 (3.2–3.4), tarsus 0.34–0.35/0.04–0.05 (6.80–8.75). Leg IV: trochanter 0.16–0.18/0.11–0.12 (1.45–1.50), femoropatella 0.54–0.55/0.18–0.21 (2.62–3.00), tibia 0.33–0.35/0.08–0.09 (3.89–4.12), basitarsus 0.16/0.06 (2.67), telotarsus 0.39/0.04 (9.75).

##### Remarks.

*Lagynochthoniushepingensis* sp. nov. most closely resembles *L.tonkinensis* in the presence of intercalary teeth on both chelal fingers and the presence of four setae on both tergal chaetotaxy I–II. However, it differs by the presence of a hump-shaped epistome and four well-developed eyes, whereas *L.tonkinensis* has a flat, rounded epistome and spot-like posterior pair of eyes (Beier 1951).

##### Distribution.

China (Guizhou Province).

#### ﻿ Lagynochthonius houi sp. nov.

Taxon classificationAnimaliaPseudoscorpionesChthoniidae

225294AA-1821-5256-98E8-9C98A9637914

https://zoobank.org/B0877E10-C978-48E8-8F96-6274F4E0BFD7

**Figure 14.**
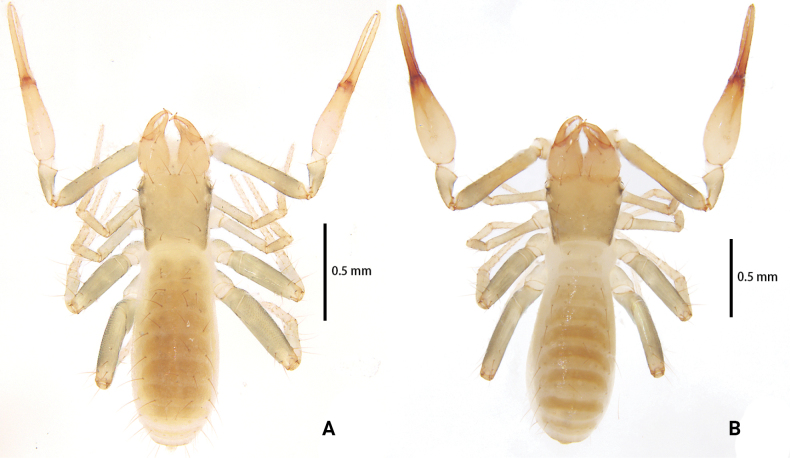
*Lagynochthoniushoui* sp. nov. **A** holotype male (dorsal view) **B** paratype female (dorsal view).

**Figure 15.**
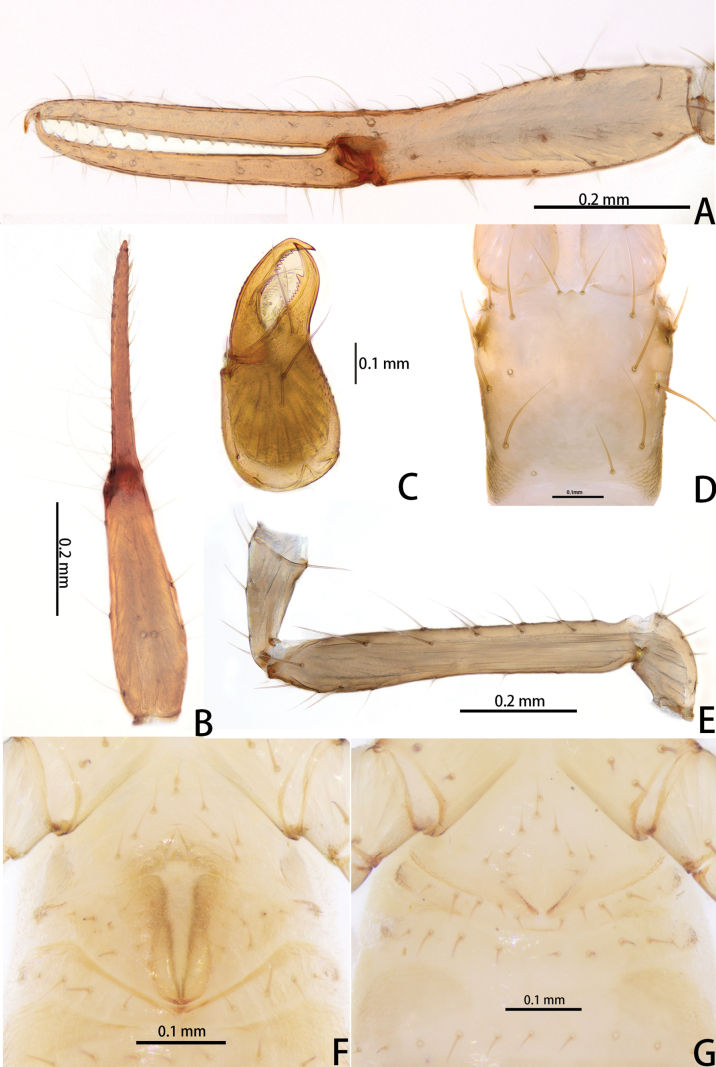
*Lagynochthoniushoui* sp. nov., holotype male (**A–F**) paratype female (**G**): **A** left chela (lateral view) **B** left chela (dorsal view) **C** left chelicera (dorsal view) **D** carapace (dorsal view) **E** left pedipalp (minus chela, dorsal view) **F** male genital area (ventral view) **G** female genital area (ventral view).

**Figure 16.**
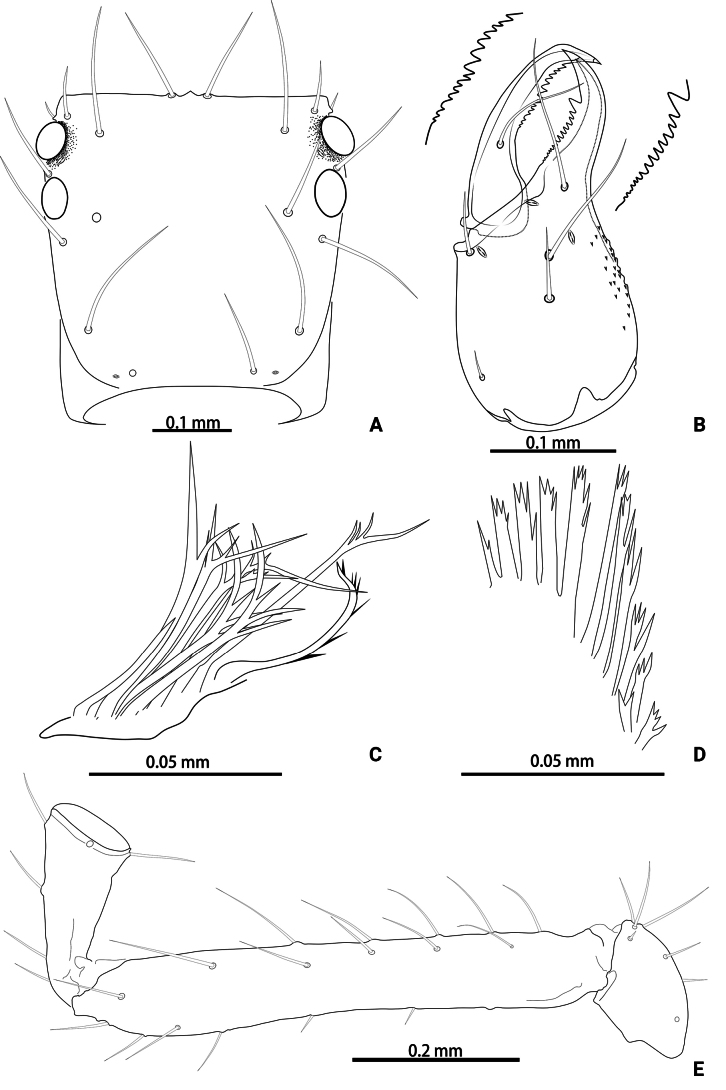
*Lagynochthoniushoui* sp. nov., holotype male **A** carapace (dorsal view) **B** left chelicera (dorsal view), with details of teeth **C** rallum **D** coxal spines on coxae II (ventral view) **E** left pedipalp (minus chela, dorsal view).

**Figure 17.**
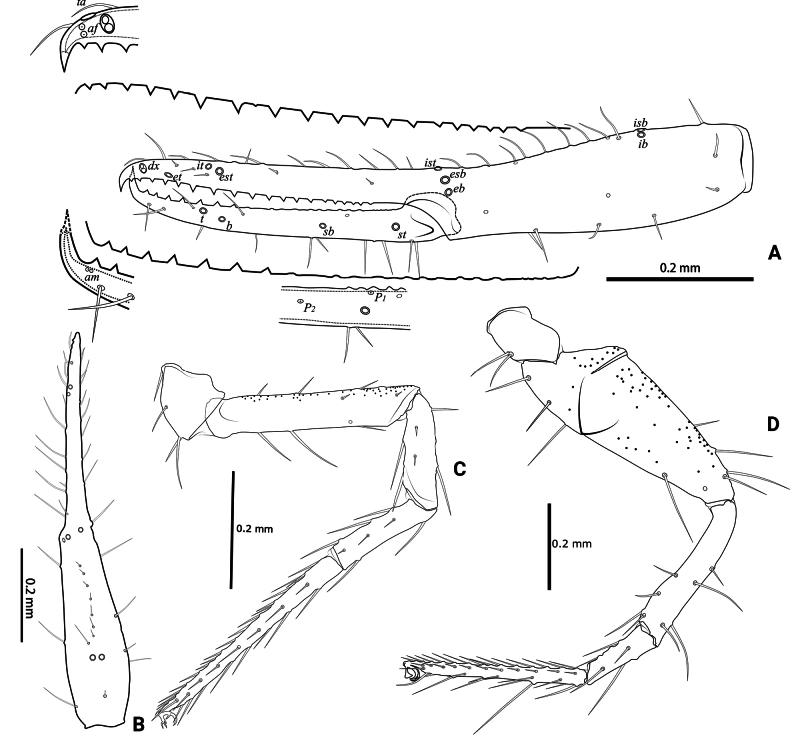
*Lagynochthoniushoui* sp. nov., holotype male **A** left chela (lateral view), with details of teeth and trichobothrial pattern **B** left chela (dorsal view) **C** leg I (lateral view) **D** leg IV (lateral view). Abbreviations: for the chelal trichobothria: *b* = basal; *sb* = sub-basal; *st* = subterminal; *t* = terminal; *ib* = interior basal; *isb* = interior sub-basal; *ist* = interior sub-terminal; *it* = interior terminal; *eb* = exterior basal; *esb* = exterior sub-basal; *est* = exterior sub-terminal; *et* = exterior terminal. For other abbreviations: *af*, apical sensilla of fixed chelal finger, *am*, apical sensilla of movable chelal finger; *dx*, duplex trichobothria; *p_1–2_*, proximal sensilla of movable chelal finger; *td*, modified tooth.

Figs 14 , 15 , 16 , 17 Chinese name: 侯氏拉伪蝎

##### Type material.

***Holotype*** ♂ (Ps.-MHBU-GZ2022080901): China, Guizhou Province, Qiannan Prefecture, Pintang County, Tangbian Town, Xindian Village, under topsoil and in the leaf litter layer [25°37′42.19″N, 106°43′55.15″E], 991 m a.s.l., 9 August 2022, Yanmeng Hou, Lu Zhang, Jianzhou Sun & Wenlong Fan leg. ***Paratypes***: 2 ♂ (Ps.-MHBU-GZ2022080902–03) and 1 ♀ (Ps.-MHBU-GZ2022080904), all with the same data as the holotype.

##### Etymology.

This species is named for Yanmeng Hou, who participated in field work and collected some of the specimens. A noun in apposition.

##### Diagnosis.

(♂♀). Moderately sized epigean species; carapace with four eyes, anterior margin smooth and epistome triangular; tergites I and II each with two setae, III and IV each with four setae. Rallum with eight blades. Pedipalps slender, chela 6.31–6.75 (♂), 5.20 (♀) × as long as broad; femur 5.90–6.78 (♂), 6.25 (♀) × as long as broad; chelal fingers without intercalary teeth, fixed chelal finger with a modified accessory tooth (*td*) on prolateral-retrolateral face; chemosensory setae (*sc*) present on dorsum of chelal hand; sensilla present.

##### Description.

**Males** (holotype and paratypes) (Figs 14A, 15A–F, 16, 17).

***Color*** generally pale yellow, chelicerae, carapace, pedipalps and tergites slightly darker.

***Cephalothorax*** (Figs 15D, 16A): carapace nearly subquadrate, 0.91–0.97 × as long as broad, weakly constricted basally; posterior region with squamous sculpturing laterally, other aera smooth, without furrows; anterior margin smooth, without serrate; epistome small and triangular; four eyes, anterior pair of eyes well-developed, posterior pair with flat lenses; with 18 setae arranged s4s: 4: 4: 2: 2, most setae heavy, long and gently curved, anterolateral setae much shorter than others; with two pairs of lyrifissures, first pair situated middle to the setae of ocular row, second pair situated lateral to the sole pair of setae of posterior row. Manducatory process with two acuminate distal setae, anterior seta less than 1/2 length of medial seta; apex of coxa I with a rounded anteromedial process; coxae II with 9–12 terminally indented coxal spines on each side, set as an oblique and arc row, central spines slightly longer than the others (Fig. 16D); intercoxal tubercle absent; Chaetotaxy of coxae: P 3, I 3, II 4, III 5, IV 5.

***Chelicera*** (Figs 15C, 16B): almost as long as carapace, 1.71–1.88 × as long as broad; five setae and three lyrifissures (including an exterior condylar lyrifissure, an exterior lyrifissure and extra lyrifissure (near sub basal setae)) present on hand, all setae acuminate, ventrobasal setae shorter than others; movable finger with one medial seta. Cheliceral palm has moderate wrinkle on both ventral and dorsal sides. Both fingers well provided with teeth, fixed finger with 9–12 teeth, distal one largest; movable finger with 11–13 retrorse contiguous small teeth; galea completely vestigial (Fig. 16B). Serrula exterior with 17–22 and serrula interior with 11–14 blades. Rallum with eight blades, the distal one longest and recumbent basally, with fine barbules and slightly set apart from the other blades, latter tightly grouped and with long pinnae, some of which are subdivided (Fig. 16C).

***Pedipalp*** (Figs 15A, B, E, 16E, 17A, B): trochanter 2.00–2.12, femur 5.90–6.78, patella 2.44–2.67, chela 6.31–6.75, hand 3.23–3.25 × as long as broad; femur 2.45–2.68 × as long as patella; movable chelal finger 0.95–1.05 × as long as hand and 0.49–0.51 × as long as chela. Setae generally long and acuminate. Chelal palm gradually constricted towards fingers, apodeme complex of movable chelal finger strongly sclerotized. Fixed chelal finger and hand with eight trichobothria, movable chelal finger with four trichobothria, *ib* and *isb* situated close together, submedially on dorsum of chelal hand; *eb*, *esb* and *ist* forming a straight oblique row at base of fixed chelal finger; *it* slightly distal to *est*, situated subdistally; *et* slightly near to tip of fixed chelal finger, very close to chelal teeth; *dx* situated distal to *et*; *sb* slightly closer to *st* than to *b*; *b* and *t* situated subdistally, *t* situated at the same level as *it* and distal to *b*; *est* situated distal to *b* (Figs 15A, 17A). Fixed chelal finger with sensilla *af_1–2_* close together, near tip; movable chelal finger with four sensilla: *am_1–2_* near tip, *p_2_* slightly distad of *sb*, *p_1_* distad of *p_2_* and very close to chelal teeth (Fig. 17A). Microsetae (chemosensory setae) present on dorsum of chelal hand (Figs 15B, 17B). Both chelal fingers with a row of teeth, spaced regularly along the margin, teeth smaller distally and proximally: fixed finger with 15–19 well-spaced, pointed teeth, and a modified accessory tooth on prolateral-retrolateral face (*td*, slightly distal to *dx*); movable finger with seven well-spaced, pointed teeth, plus 10–12 vestigial, rounded and contiguous basal teeth.

***Opisthosoma***: generally typical, pleural membrane finely granulated. All tergites and sternites undivided; setae uniseriate and acuminate. Tergal chaetotaxy I–XII: 2: 2: 4: 4: 4: 4: 4: 5–6: 5–8: 4: T2T: 0. Sternal chaetotaxy IV–XII: 10: 10–11: 8–10: 8–10: 9–10: 9–10: 9: -: 2. Genital region: sternite II with 8–9 setae scattered on median area, genital opening slit-like, sternite III with 16–18 setae (Fig. 15F).

***Legs*** (Fig. 17C, D): fine granulation present on anterodorsal faces of trochanter IV, femur I, IV and patella IV. Leg III: Femur 1.94–2.21 × as long as patella; tarsus 2.00–2.29 × as long as tibia. Leg IV: Femoropatella 2.67–3.29 × as long as deep; tibia 5.00–5.83 × as long as deep; with basal tactile setae on both tarsal segments: Basitarsus 2.67–3.60 × as long as deep (TS = 0.31–0.39), telotarsus 10.50–13.00 × as long as deep and 2.47–2.50 × as long as basitarsus (TS = 0.23–0.26). Setae of leg I (trochanter to tibia) 3–4: 9–10: 4–6: 6–8, setae of leg IV (trochanter to basitarsus) 2–3: 2–3: 4–6: 7–8: 5–7. Arolium not divided, slightly shorter than the simple claws.

**Adult female** (paratype) (Figs 14B, 15G). mostly same as males; tergal chaetotaxy I–XII: 2: 2: 4: 4: 4: 5: 5: 6: 6: 4: T2T: 0; sternal chaetotaxy IV–XII: 8: 11: 10: 10: 10: 11: 9: -: 2. Genital region: sternite II with 10 setae scattered on median area, sternite III with a row of 10 setae.

***Dimensions*** (length/breadth or, in the case of the legs, length/depth in mm; ratios in parentheses). Males: Body length 1.29–1.41. Pedipalps: trochanter 0.17–0.18/0.08–0.09 (2.00–2.12), femur 0.59–0.61/0.09–0.10 (5.90–6.78), patella 0.22–0.24/0.09–0.10 (2.30–2.67), chela 0.77–0.82/0.12–0.13 (6.31–6.75), hand 0.39–0.42/0.12–0.13 (3.23–3.25), movable chelal finger length 0.38–0.41. Chelicera 0.29–0.32/0.17 (1.71–1.88), movable finger length 0.17–0.19. Carapace 0.32–0.36/0.35–0.37 (0.91–0.97). Leg I: trochanter 0.11–0.12/0.07–0.09 (1.33–1.57), femur 0.31–0.35/0.07–0.09 (5.50–6.20), patella 0.14–0.17/0.04–0.05 (2.80–4.25), tibia 0.16–0.18/0.04 (4.00–4.50), tarsus 0.36–0.39/0.03–0.04 (9.75–12.67). Leg IV: trochanter 0.17–0.19/0.10–0.13 (1.31–1.72), femoropatella 0.54–0.56/0.17–0.21 (2.67–3.29), tibia 0.35–0.37/0.06–0.07 (5.00–5.83), basitarsus 0.16–0.18/0.05–0.06 (2.67–3.60), telotarsus 0.39–0.42/0.03–0.04 (10.50–13.00).

**Females**: body length 1.68. Pedipalps: trochanter 0.22/0.11 (2.00), femur 0.75/0.12 (6.25), patella 0.28/0.13(2.15), chela 1.04/0.20 (5.20), hand 0.55/0.12 (2.75), movable chelal finger length 0.50. Chelicera 0.41/0.24 (1.71), movable finger length 0.25. Carapace 0.40/0.47 (0.85). Leg I: trochanter 0.13/0.12 (1.08), femur 0.40/0.08 (5.00), patella 0.21/0.07 (3.00), tibia 0.21/0.06 (3.50), tarsus 0.46/0.05 (9.20). Leg IV: trochanter 0.21/0.14 (1.50), femoropatella 0.67/0.25 (2.68), tibia 0.43/0.08 (5.38), basitarsus 0.22/0.07 (3.14), telotarsus 0.51/0.05 (10.20).

##### Remarks.

*Lagynochthoniushoui* sp. nov. differs from all other epigean species of the genus *Lagynochthonius* from China except *L.duo* sp. nov. by the following combination of characters: the presence of a triangular epistome and the presence of two setae on tergite I and II (Beier 1951, 1967; Hu and Zhang 2012a, b; Zhang and Zhang 2014).

*Lagynochthoniushoui* sp. nov. differs from *L.duo* sp. nov. in the length of the movable chelal finger which is 0.95–1.05 × as long as the hand in males and 0.91 × as long as the hand in female, whereas in *L.duo* sp. nov. it is 1.11–1.27 × as long as the hand in males and 1.11 × as long as hand in female. Additionally, *L.houi* sp. nov. lacks intercalary tooth on the chelal fingers, whereas the fixed chelal finger posesses intercalary teeth in *L.duo* sp. nov.

##### Distribution.

China (Guizhou Province).

#### ﻿ Lagynochthonius sanhuaensis sp. nov.

Taxon classificationAnimaliaPseudoscorpionesChthoniidae

B02CFA2D-BD87-56B1-8D6F-77FCB54F1FD9

https://zoobank.org/0A34AF68-B0BD-4BBA-BB44-A98AB2F32872

**Figure 18.**
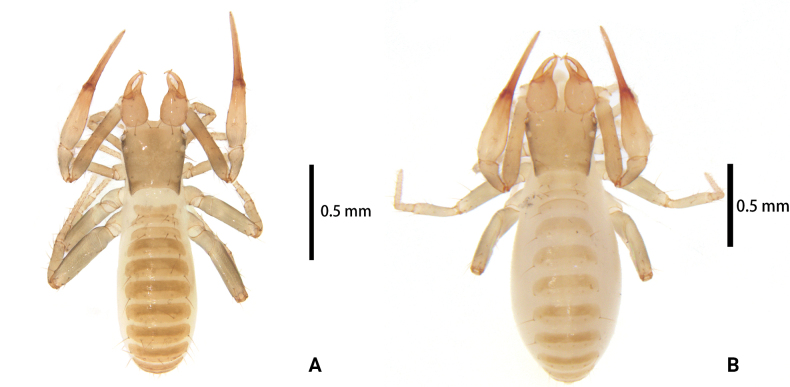
*Lagynochthoniussanhuaensis* sp. nov. **A** holotype male (dorsal view) **B** paratype female (dorsal view).

**Figure 19.**
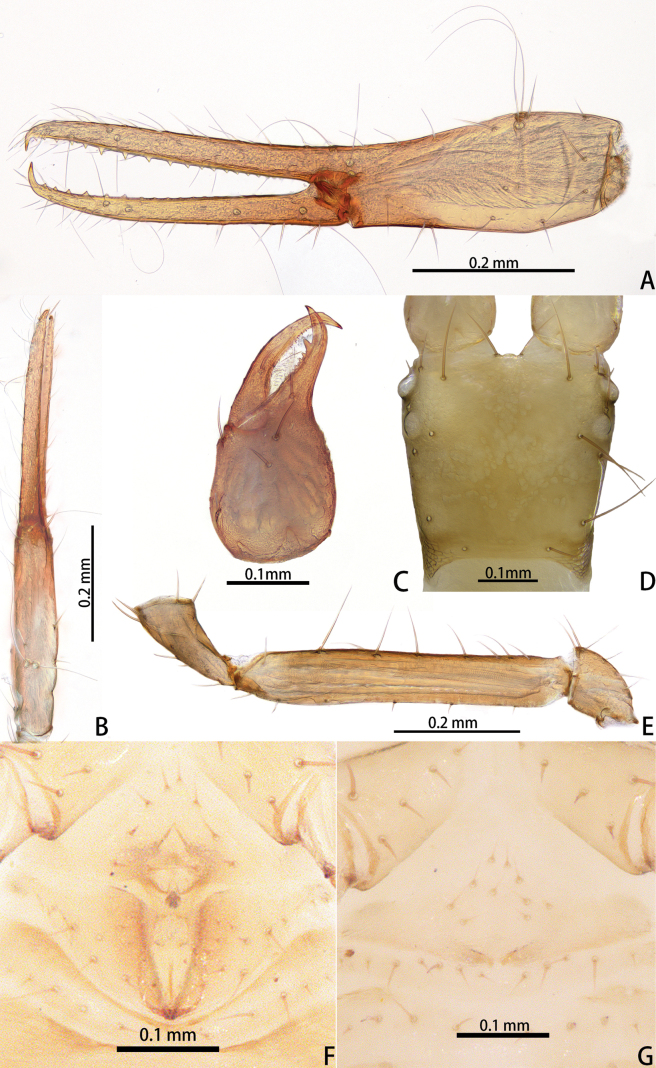
*Lagynochthoniussanhuaensis* sp. nov., holotype male (**A–F**) paratype female (**G**): **A** left chela (lateral view) **B** left chela (dorsal view) **C** left chelicera (dorsal view) **D** carapace (dorsal view) **E** left pedipalp (minus chela, dorsal view) **F** male genital area (ventral view) **G** female genital area (ventral view).

**Figure 20.**
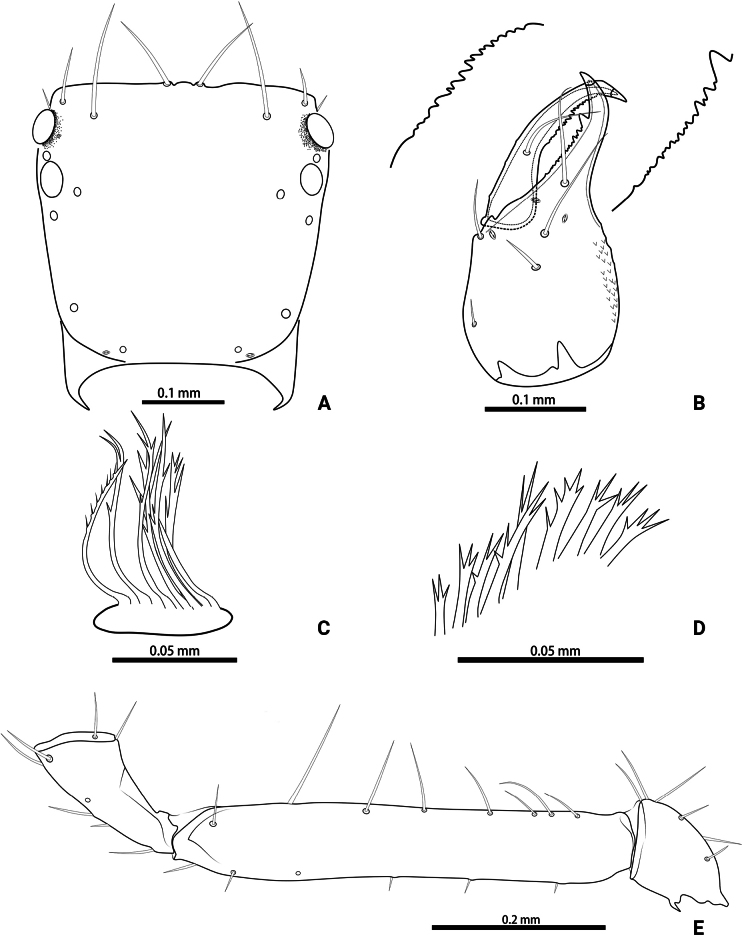
*Lagynochthoniussanhuaensis* sp. nov., holotype male **A** carapace (dorsal view) **B** left chelicera (dorsal view), with details of teeth **C** rallum **D** coxal spines on coxae II (ventral view) **E** left pedipalp (minus chela, dorsal view).

**Figure 21.**
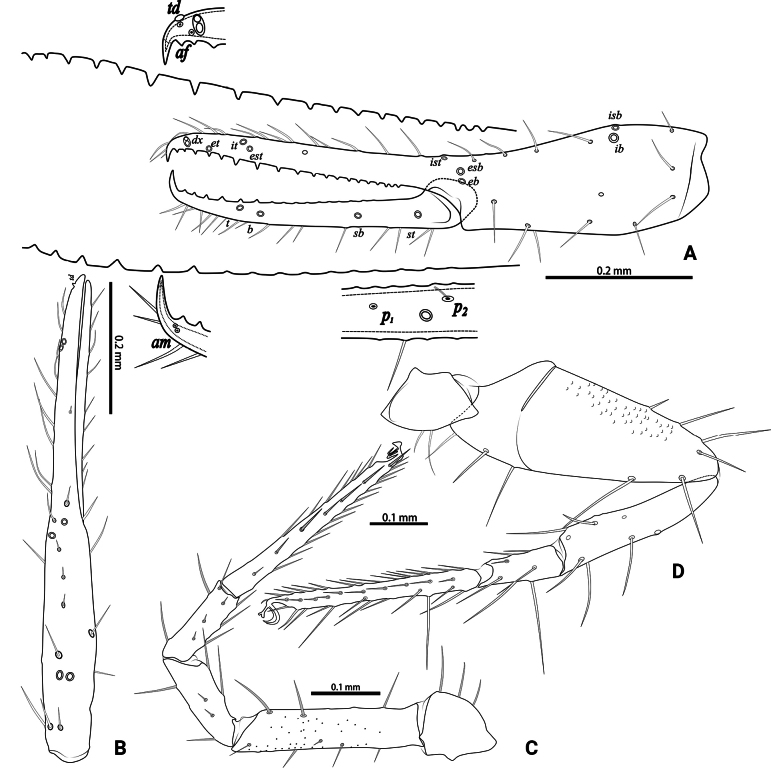
*Lagynochthoniussanhuaensis* sp. nov., holotype male **A** left chela (lateral view), with details of teeth and trichobothrial pattern **B** left chela (dorsal view) **C** leg I (lateral view) **D** leg IV (lateral view). Abbreviations: for the chelal trichobothria: *b* = basal; *sb* = sub-basal; *st* = subterminal; *t* = terminal; *ib* = interior basal; *isb* = interior sub-basal; *ist* = interior sub-terminal; *it* = interior terminal; *eb* = exterior basal; *esb* = exterior sub-basal; *est* = exterior sub-terminal; *et* = exterior terminal. For other abbreviations: *af*, apical sensilla of fixed chelal finger, *am*, apical sensilla of movable chelal finger; *dx*, duplex trichobothria; *p_1–2_*, proximal sensilla of movable chelal finger; *td*, modified tooth.

Figs 18 , 19 , 20 , 21 Chinese name: 三花拉伪蝎

##### Type material.

***Holotype*** ♂ (Ps.-MHBU-GZ2022070201): China, Guizhou Province, Tongren City, Yinjiang County, 500 m near Sanhua Mountain, under topsoil and in the leaf litter layer [27°53′40.73″N, 108°32′46.16″E], 818 m a.s.l., 7 July 2022, Yanmeng Hou, Lu Zhang, Nana Zhan, Jianzhou Sun & Long Lin leg. ***Paratypes***: 2 ♂ (Ps.-MHBU-GZ2022070203 & GZ2022070205) and 2 ♀ (Ps.-MHBU-GZ2022070202 & GZ2022070204), all with the same data as the holotype.

##### Etymology.

Named after the Sanhua Mountain, near the type locality. A noun in apposition.

##### Diagnosis.

(♂♀). Moderately sized epigean species; carapace with four eyes, anterior margin smooth and epistome hump-shaped; tergites I–IV each with four setae. Rallum with seven blades. Pedipalps slender, chela 6.80–7.89 (♂), 5.31–5.40 (♀) × as long as broad; femur 6.50–6.63 (♂), 5.70–6.00 (♀) × as long as broad; chelal fingers without intercalary teeth, fixed chelal finger with a modified accessory tooth (*td*) on prolateral-retrolateral face; chemosensory setae (*sc*) present on dorsum of chelal hand; sensilla present.

##### Description.

**Males** (holotype and paratypes) (Figs 18A, 19A–F, 20, 21).

***Color*** generally pale yellow, chelicerae, carapace, pedipalps and tergites slightly darker.

***Cephalothorax*** (Figs 19D, 20A): carapace nearly subquadrate, 0.92–0.97 × as long as broad, weakly constricted basally; posterior region with squamous sculpturing laterally, other area smooth, without furrows; anterior margin smooth, without serrate; epistome small and hump-shaped; four eyes, anterior pair of eyes well-developed, posterior pair with flat lenses; with 18 setae arranged s4s: 4: 4: 2: 2, most setae heavy, long and gently curved, anterolateral setae much shorter than others; with two pairs of lyrifissures, first pair situated middle to the setae of ocular row, second pair situated lateral to the sole pair of setae of posterior row. Manducatory process with two acuminate distal setae, anterior seta less than 1/2 length of medial seta; apex of coxa I with a rounded anteromedial process; coxae II with 9–11 terminally indented coxal spines on each side, set as an oblique and arc row, central spines slightly longer than the others (Fig. 20D); intercoxal tubercle absent; Chaetotaxy of coxae: P 3, I 3, II 4, III 5, IV 5.

***Chelicera*** (Figs 19C, 20B): almost as long as carapace, 1.81–1.93 × as long as broad; five setae and three lyrifissures (including an exterior condylar lyrifissure, an exterior lyrifissure and extra lyrifissure (near sub basal setae)) present on hand, all setae acuminate, ventrobasal setae shorter than others; movable finger with one medial seta. Cheliceral palm has moderate wrinkle on both ventral and dorsal sides. Both fingers well provided with teeth, fixed finger with 14–18 teeth, distal one largest; movable finger with 16–18 retrorse contiguous small teeth; galea completely vestigial (Fig. 20B). Serrula exterior with 19–20 and serrula interior with 10–12 blades. Rallum with seven blades, the distal one longest and recumbent basally, with fine barbules and slightly set apart from the other blades, latter tightly grouped and with long pinnae, some of which are subdivided (Fig. 20C).

***Pedipalp*** (Figs 19A, B, E, 20E, 21A, B): trochanter 1.88–2.14, femur 6.50–6.75, patella 2.22–2.50, chela 6.80–7.89, hand 3.20–3.67 × as long as broad; femur 2.57–2.65 × as long as patella; movable chelal finger 1.15–1.19 × as long as hand and 0.53–0.56 × as long as chela. Setae generally long and acuminate. Chelal palm gradually constricted towards fingers, apodeme complex of movable chelal finger strongly sclerotized. Fixed chelal finger and hand with eight trichobothria, movable chelal finger with four trichobothria, *ib* and *isb* situated close together, submedially on dorsum of chelal hand; *eb*, *esb* and *ist* at base of fixed chelal finger; *esb* and *eb* at almost the same level and *ist* slightly distal to *esb*; *it* slightly distal to *est*, situated subdistally; *et* slightly near to tip of fixed chelal finger, very close to chelal teeth; *dx* situated distal to *et*; *sb* slightly closer to *st* than to *b*; *b* and *t* situated subdistally, *t* situated at the same level as *it* and distal to *b*; *est* situated distal to *b* and close to *it* (Figs 19A, 21A). Fixed chelal finger with sensilla *af_1–2_* close together, near tip; movable chelal finger with four sensilla: *am_1–2_* near tip, *p_2_* slightly distad of *sb*, *p_1_* proximad of *sb* and very close to chelal teeth (Fig. 21A). Microsetae (chemosensory setae) present on dorsum of chelal hand (Figs 19B, 21B). Both chelal fingers with a row of teeth, spaced regularly along the margin, teeth smaller distally and proximally: fixed finger with 18 or 19 well-spaced, pointed teeth, and a modified accessory tooth on prolateral-retrolateral face (*td*, slightly distal to *dx*); movable finger with six well-spaced, pointed teeth, plus 8–10 vestigial, rounded and contiguous basal teeth.

***Opisthosoma***: generally typical, pleural membrane finely granulated. All tergites and sternites undivided; setae uniseriate and acuminate. Tergal chaetotaxy I–XII: 4: 4: 4: 4: 4: 4–6: 4–6: 5–6: 6: 4: T2T: 0. Sternal chaetotaxy IV–XII: 10–12: 10–11: 11–13: 9–13: 10–12: 10–12: 9: -: 2. Genital region: sternite II with ten setae scattered on median area, genital opening slit-like, sternite III with 16–18 setae (Fig. 19F).

***Legs*** (Fig. 21C, D): fine granulation present on anterodorsal faces of trochanter IV, femur I; scale-like texture display on anterodorsal faces of femoropatella IV. Leg I: femur 2.57–2.65 × as long as patella; tarsus 2.07–2.28 × as long as tibia. Leg IV: femoropatella 2.67–3.00 × as long as deep; tibia 4.43–5.33 × as long as deep; with basal tactile setae on both tarsal segments: basitarsus 2.50–3.50 × as long as deep (TS = 0.36–0.43), telotarsus 8.75–11.67 × as long as deep and 2.33–2.36 × as long as basitarsus (TS = 0.21–0.23). Setae of leg I (trochanter to tibia) 3–4: 7–8: 3–6: 6–9, setae of leg IV (trochanter to basitarsus) 2–3: 3–5: 5–6: 7–8: 5–7. Arolium not divided, slightly shorter than the simple claws.

**Adult females** (paratypes) (Figs 18B, 19G). Mostly same as males; tergal chaetotaxy I–XII: 4: 4: 4: 4: 4–6: 6: 5–6: 6: 6: 4: T2T: 0; sternal chaetotaxy IV–XII: 12–13: 13–15: 11–13: 11–12: 12: 11: 9–10: -: 2. Genital region: sternite II with ten setae scattered on median area, sternite III with a row of 10–12 setae.

***Dimensions*** (length/breadth or, in the case of the legs, length/depth in mm; ratios in parentheses). Males: body length 1.30–1.36. Pedipalps: trochanter 0.15/0.07–0.08 (1.88–2.14), femur 0.52–0.54/0.08 (6.50–6.63), patella 0.20–0.21/0.08–0.09 (2.22–2.50), chela 0.68–0.73/0.09–0.10 (6.80–7.89), hand 0.32–0.34/0.09–0.10 (3.20–3.67), movable chelal finger length 0.38–0.41. Chelicera 0.29–0.30/0.15–0.16 (1.81–1.93), movable finger length 0.18–0.19. Carapace 0.32–0.36/0.35–0.37 (0.91–0.97). Leg I: trochanter 0.09–0.11/0.08–0.09 (1.00–1.22), femur 0.27–0.28/0.05 (5.40–5.60), patella 0.13–0.15/0.05–0.06 (2.33–3.00), tibia 0.14–0.15/0.04–0.05 (2.80–3.75), tarsus 0.30–0.32/0.03 (10.00–10.67). Leg IV: trochanter 0.13–0.16/0.10–0.11 (1.30–1.45), femoropatella 0.46–0.48/0.16–0.18 (2.67–3.00), tibia 0.31–0.32/0.06–0.07 (4.43–5.33), basitarsus 0.14–0.15/0.04–0.06 (2.50–3.50), telotarsus 0.33–0.35/0.03–0.04 (8.75–11.67).

**Females**: body length 1.61–1.71. Pedipalps: trochanter 0.15–0.19/0.08–0.09 (1.67–2.38), femur 0.57–0.60/0.10 (5.70–6.00), patella 0.25–0.26/0.11 (2.27–2.36), chela 0.81–0.85/0.15–0.16 (5.2), hand 0.39–0.41/0.15–0.16 (2.75), movable chelal finger length 0.43–0.45. Chelicera 0.34–0.37/0.19–0.21 (1.76–1.79), movable finger length 0.22–0.23. Carapace 0.35–0.36/0.34–0.37 (0.82–0.88). Leg I: trochanter 0.09–0.13/0.09 (1.33–1.44), femur 0.30–0.32/0.06–0.08 (3.75–5.33), patella 0.17–0.18/0.05–0.06 (2.83–3.60), tibia 0.16–0.17/0.04–0.05 (3.20–4.25), tarsus 0.34–0.35/0.04 (8.50–8.75). Leg IV: trochanter 0.15–0.19/0.10–0.12 (1.50–1.58), femoropatella 0.49–0.54/0.21 (2.33–2.57), tibia 0.35–0.36/0.07 (5.00–5.14), basitarsus 0.15–0.16/0.06–0.07 (2.29–2.50), telotarsus 0.36–0.38/0.03–0.04 (9.50–12.00).

##### Remarks.

*Lagynochthoniussanhuaensis* sp. nov. most closely resembles *L.niger* in the absence of intercalary teeth and the presence of four setae on tergites I and II, but differs from it in several characters. First, the new species has a shorter (0.68–0.73 mm compared to 0.75–0.93 mm in *L.niger*) and thinner chela (6.80–7.89 × as long as broad compared to 5.17–6.25 × as long as broad in *L.niger*) in males. Second, the shape of the epistome is hump-shaped in *L.sanhuaensis* sp. nov., whereas it is triangular in *L.niger*. Third, the rallum has seven blades in *L.sanhuaensis* sp. nov., whereas it has eight blades in *L.niger* (Hu and Zhang 2012a).

##### Distribution.

China (Guizhou Province).

### ﻿Key to the epigean species of *Lagynochthonius* from China

**Table:** 

1	Tergites I and II each with two setae	**2**
–	Tergites I and II each with four setae	**4**
2	Epistome present	**3**
–	Epistome absent	***L.medog* Zhang & Zhang, 2014**
3	Only fixed chelal finger with intercalary teeth	***L.duo* sp. nov.**
–	Both chelal fingers without intercalary teeth	***L.houi* sp. nov.**
4	At least one finger of chela with intercalary teeth	**5**
–	Both chelal fingers without intercalary teeth	**8**
5	Both chelal fingers with intercalary teeth	**6**
–	Only fixed chelal finger with intercalary teeth	**7**
6	Carapace with a hump-shaped epistome; the four well-developed eyes	***L.hepingensis* sp. nov.**
–	Carapace with a flat, rounded epistome; posterior pair of eyes spot-like	***L.tonkinensis* (Beier, 1951)**
7	Carapace without epistome	***L.harveyi* Zhang & Zhang, 2014**
–	Carapace with a hump-shaped epistome	***L.gibbus* sp. nov.**
8	Epistome present	**9**
–	Epistome absent	**10**
9	Epistome triangular; pedipalpal chela length 0.75–0.93 mm, 5.17–6.25 × as long as broad	***L.niger* Hu & Zhang, 2012**
–	Epistome hump-shaped; pedipalpal chela length 0.68–0.73 mm, 6.80–7.30 × as long as broad	***L.sanhuaensis* sp. nov.**
10	Four well-developed eyes	**11**
–	Anterior pair of eyes well-developed, posterior pair of eyes reduced to eyespot	***L.brachydigitatus* Zhang & Zhang, 2014**
11	Pedipalpal chela length 0.72 mm, 4.80 × as long as broad	***L.sinensis* (Beier, 1967)**
–	Pedipalpal chela length 0.95–1.00 mm, 5.56–6.33 × as long as broad	***L.leptopalpus* Hu & Zhang, 2012**

## Supplementary Material

XML Treatment for Lagynochthonius

XML Treatment for Lagynochthonius duo

XML Treatment for Lagynochthonius gibbus

XML Treatment for Lagynochthonius hepingensis

XML Treatment for Lagynochthonius houi

XML Treatment for Lagynochthonius sanhuaensis

## ﻿Additional information

### Author ORCIDs

Jianzhou Sun https://orcid.org/0009-0001-4940-7130

Xiangbo Guo https://orcid.org/0000-0002-7074-8642

Feng Zhang https://orcid.org/0000-0002-3347-1031

### Data availability

All of the data that support the findings of this study are available in the main text.
